# Bioabsorbable Endovascular Adhesive Tape (BEAT) for Improving Vascular Regeneration

**DOI:** 10.1002/advs.202512857

**Published:** 2026-02-10

**Authors:** Jiarong Wang, Jing Wang, Xinyi Li, Bo Yu, Yiduo Chen, Yirong Guo, Honglin Qian, Meng Hu, Haoyang Liu, Wenhui Liu, Han Xu, Kefeng Ren, M. Cristina L. Martins, Jian Ji

**Affiliations:** ^1^ State Key Laboratory of Transvascular Implantation Devices Department of Cardiology The Second Affiliated Hospital, School of Medicine Zhejiang University Hangzhou P. R. China; ^2^ MOE Key Laboratory of Macromolecular Synthesis and Functionalization Department of Polymer Science and Engineering Zhejiang University Hangzhou P. R. China; ^3^ Transvascular Implantation Devices Research Institute Hangzhou P. R. China; ^4^ I3S‐Instituto de Investigaçāo e Inovaçāo Em Saúde Universidade Do Porto INEB‐Instituto de Engenharia Biomédica Portugal

**Keywords:** drug‐coated balloon, endothelialization, polyelectrolyte complex, tissue adhesive, vascular regeneration

## Abstract

The drug‐coated balloons (DCBs) provide a combination therapy of balloon angioplasty and anti‐proliferative drug delivery to target lesions, thereby facilitating the appealing concept of leaving nothing behind. However, several studies have highlighted significant challenges posed by low drug transfer efficiency and immediate lumen loss due to elastic recoil. Herein, we propose a bioabsorbable endovascular adhesive tape (BEAT) platform that can realize robust adhesion and complete transfer after balloon inflation, enabling efficient endoluminal drug delivery while providing temporary radial support against elastic recoil. This BEAT coated balloon contains sprayable Janus coating layers of crosslinked drug‐eluting polyelectrolyte complexes (PECs) and poly(thioctic acid) (PTA) adhesive layer. Notably, the poly (L‐lysine‐co‐L‐leucine)‐poly (acrylic acid) PECs (PKL‐PAA, KLA) exhibit exceptional anti‐coagulation properties and selective endothelial cell adhesion behavior. Moreover, the hydrophobic interaction and photo‐controllable crosslinking of KLA PECs enable flexible mechanical tunability (0.74–10.9 MPa), robust swelling resistance, and enzyme‐responsive biodegradability, thus guaranteeing temporary mechanical reinforcement for blood vessels with efficient drug delivery. In a rat abdominal aorta injury model, this BEAT coated balloon demonstrates intact transfer with mechanical compliance to the vessel and effectively attenuates neointimal hyperplasia. This BEAT platform offers a promising perspective for the development of DCBs in vascular interventions.

## Graphical abstract

A bioabsorbable endovascular adhesive tape (BEAT) platform based on a hierarchical polyelectrolyte complex is developed to enhance drug delivery in the DCB system. The coating enables rapid transfer to the vessel, provides short‐term mechanical support, and promotes both immediate transfer and long‐term retention of drugs at the lesion site. This approach shows significant potential for advancing balloon‐mediated clinical interventions.

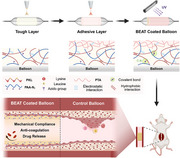

## 1 Introduction

Vascular diseases, including myocardial infarction, stroke, coronary artery disease (CAD), and peripheral artery disease (PAD), are the leading cause of global mortality [1, 2]. Currently, percutaneous coronary intervention (PCI) with implantation of a drug‐eluting stent (DES) is the most prevalent procedure for CAD [3]. However, clinical data reveal a significant concern regarding late stent thrombosis and restenosis with an increasing proportion of nearly 2% per year after implantation [4, 5, 6, 7]. Alternatively, the drug‐coated balloon (DCB) provides a combination therapy of balloon angioplasty and anti‐proliferative drugs to treat targeted lesions [8, 9, 10, 11, 12]. This “leaving nothing behind” strategy is promising to avoid stent‐induced thrombosis and achieve lower restenosis rates [13, 14, 15]. Besides, DCB takes advantage in the treatment of several series of lesions, such as small and blanched vessels, revascularization of previously stented vessels, etc. [15, 16, 17]. Despite the promising benefits, the successful DCB therapy requires sufficient and uniform delivery of anti‐proliferative drugs during the short intervention duration. Hydrophobic drugs, such as paclitaxel (PTX) with a micro‐sized sharp crystalline structure, are currently employed to improve the uptake rate and release profile [14, 18, 19, 20]. However, several studies have indicated relatively low transfer efficiency and immediate lumen loss due to elastic recoil, which poses significant challenges to the therapeutic effect and safety concerns [13, 21, 22]. Developing a novel DCB system with enhanced drug delivery efficiency and improved resistance to vessel recoil is of great significance for clinical practice in balloon‐based PCI.

Commercial drug excipients (urea, iopromide, phospholipid, etc.) are designed to promote drug‐coating integration during fabrication, while the rapid solubility in blood results in the premature drug detachment [9, 23, 24]. In contrast to small‐molecular excipients, polymer‐based excipients have demonstrated superior advantages in research fields, including tunable solubility, toughness, and compatibility with different functional species [25]. For instance, a pioneering study by Lynn et al. introduced polyelectrolyte multilayers (PEMs) for delivering plasmid DNA to vascular tissue in vivo and suggested the great potential of balloon‐mediated gene therapy with reduced restenosis and improved vascular healing [26, 27]. Besides, synthetic polymers such as poly(ethylene oxide) and polyvinylpyrrolidone have been studied to modulate the drug‐coating morphology while reducing drug loss during balloon tracking [28, 29]. Although polymer‐based excipients display remarkable drug preservation capacity, the weak physical interaction with blood vessels remains a major bottleneck for the drug retention in the blood flow. More recently, hydrogel‐based bioadhesives have emerged as an innovative branch of tissue regenerative materials, facilitating numerous applications including hemostasis, wound sealing, biosensing, and flexible electronics [30, 31, 32, 33, 34]. Nevertheless, the dynamic blood flow and dense glycocalyx layer on the endothelium pose substantial obstacles to intravascular adhesion [35, 36]. In general, the ideal drug coating for a DCB system should meet several criteria: (i) The coating materials are able to repel interfacial water and resist swelling in blood flow; (ii) After being transferred to the blood vessel, the drug coating can provide temporary radial support with a robust anti‐coagulation effect, and achieve appropriate degradation during the vascular regeneration; (iii) The fabrication process for drug coating should be controllable and time‐efficient, avoiding toxic monomers.

**SCHEME 1:**
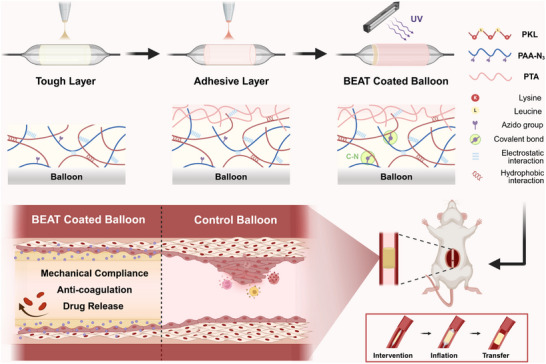
Schematic illustration of the preparation and application of a bioabsorbable endovascular adhesive tape (BEAT) platform. This BEAT coating is fabricated by sequential deposition of a KLA tough layer and a poly(thioctic acid) (PTA) adhesion layer through ultrasonic spraying. The incorporation of photo‐responsive covalent bonds and hydrophobic interactions significantly enhances the mechanical strength of KLA coating and facilitates drug loading. During the balloon intervention procedure, the BEAT coating rapidly adheres to the inner wall of the blood vessel. In contrast to conventional balloon interventions, this BEAT‐coated balloon provides instant mechanical support with dynamic compliance, enhanced anticoagulant properties, and a reduced inflammatory response, thereby promoting vascular regeneration.

Owing to excellent biocompatibility and flexible matrix binding capacity, polyelectrolyte complex (PEC) materials have garnered tremendous interest in biomedical fields for constructing extracellular mimicking surfaces on diverse medical devices, such as cardiovascular stents and bone implants [37, 38, 39, 40, 41, 42]. Given the dynamic mobility of a charged assembly, PECs have demonstrated the ability to diffuse into various tissues for achieving strong wet adhesion [43]. We recently introduced a sprayable PEC coating platform and achieved effective delivery of miRNAs for regulating vascular regeneration [25, 44]. However, the high water affinity of polyelectrolytes results in poor compatibility with most hydrophobic drugs, thereby limiting drug loading capacity and fabrication efficiency. Besides, the water‐induced plasticization of charged assemblies may further lead to decreased mechanical strength and undesired coating detachment from the vascular wall. To overcome these limitations, we introduce hydrophobic segments into a positively‐charged polypeptide (composed of L‐lysine and L‐leucine, PKL), and propose a bioabsorbable endovascular adhesive tape (BEAT) platform (Scheme 1) for promoting drug delivery of DCB system based on a hierarchical polyelectrolyte complex: (i) The cationic PKL and photoreactive anionic poly(acrylic acid) (PAA) derivative (PAA‐N_3_) are sprayed onto the balloon surface to form a mechanical layer (KLA coating); (ii) poly(thioctic acid) (PTA), which exhibits strong tissue adhesion [45, 46], is subsequently sprayed to create an adhesive layer. By controlling the ratio of PKL to PAA in the KLA coating, we optimized the anti‐coagulation performance and facilitated the competitive regeneration of endothelial cells. Upon ultraviolet (UV) irradiation, the azide groups rapidly formed covalent bonds to establish a crosslinked network, significantly enhancing the mechanical strength of the coating with a Young's modulus of up to 10.3 MPa under wet conditions. Besides, the presence of hydrophobic groups on PKL enabled efficient loading and sustained release of hydrophobic drugs. In a rat abdominal aorta injury model, the in vivo benefits of this BEAT coated balloon were further demonstrated by rapid transfer, short‐term mechanical support, and inhibition of restenosis. This strategy is of great significance for the clinical application of balloon‐based interventions.

## 2 Results and Discussion

### 2.1 Synthesis and Preparation of PKL‐PAA (KLA) Polyplex

**FIGURE 1:**
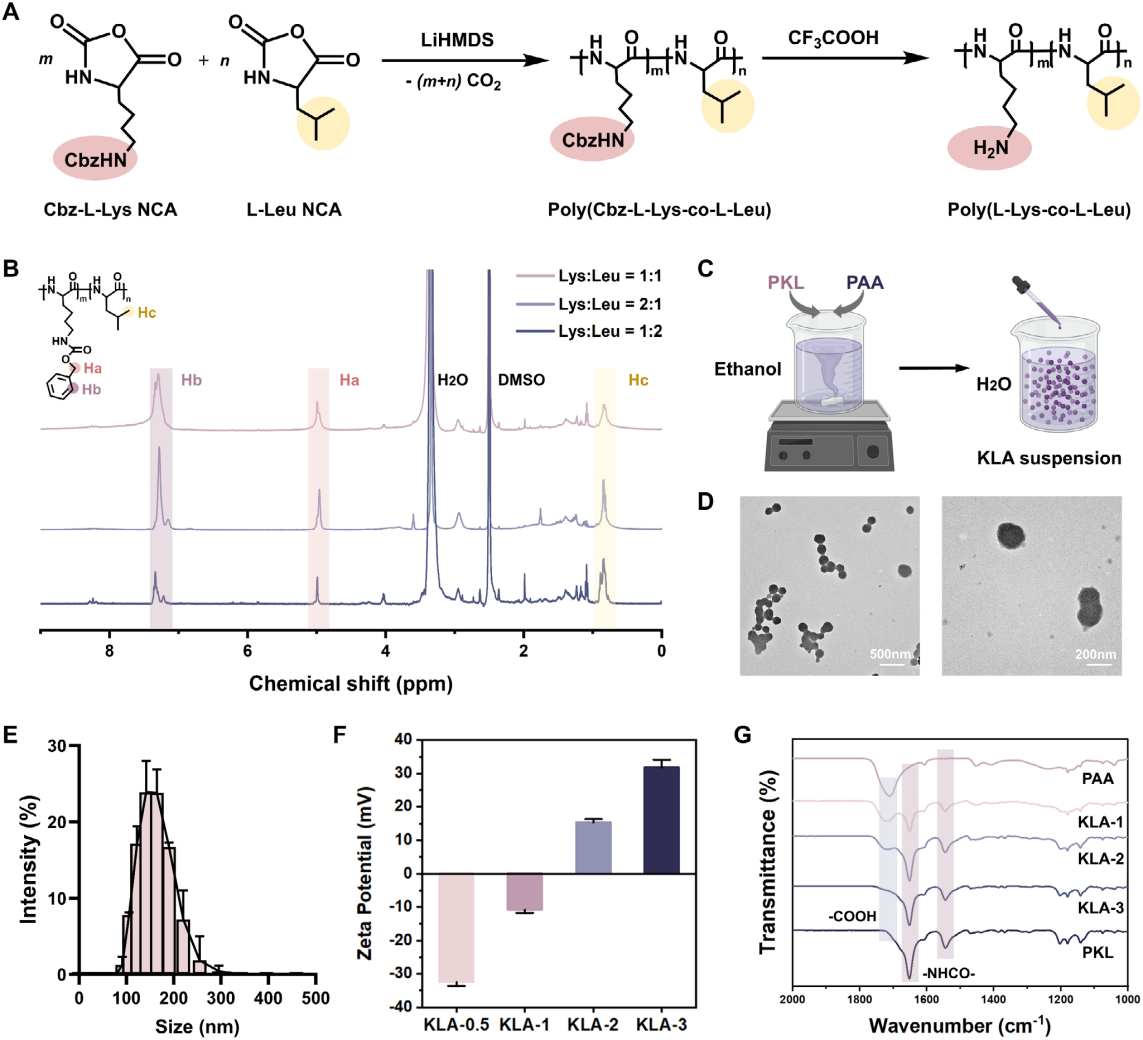
The preparation of PKL‐PAA (KLA) polyplex: (A) Schematic diagram of the synthesis of PKL. (B) ^1^H NMR spectra of PKL synthesized with different K/L ratios. (C) Schematic diagram of the preparation of the KLA polyplex suspension. Created with BioRender.com. TEM micrographs (Scale bars: 500 and 200 nm) (D), particle size distribution analysis (*n* = 3) (E), zeta potential measurements (*n* = 3) (F), and FTIR spectra (G) of KLA nanoparticles prepared with different PKL/PAA ratios. Data were presented as mean ± SD.

To achieve optimal biodegradability, we selected poly(L‐lysine‐co‐L‐leucine) polypeptide (PKL) as a polycation to construct polyelectrolyte complexes. Based on previous work, we adopted ring‐opening polymerization to synthesize the PKL peptide (Figure 1A) [47]. The lysine groups provide positive charges, while the hydrophobic side chains of leucine enhance the swelling resistance of the polyelectrolyte complexes. The chemical structure and molecular weight of PKL were confirmed by the ^1^H NMR spectroscopy and gel permeation chromatography (GPC). As illustrated in Figure 1B, the characteristic peaks in the ^1^H NMR spectra were assigned as follows: *δ* 7.33 (q, 5H, ─CH), 4.99 (d, 2H, *─*O*─*CH_2_ *─*), 0.95–0.69 (m, 12H, *─*CH_3_). According to the characteristic peaks at 7.33 ppm and 0.95–0.69 ppm in ^1^H NMR spectra, we confirmed that the proportion of lysine and leucine groups (K/L ratio) in the polypeptides was about 0.5, 1, and 2, which was close to the monomer ratio. The molecular weights of the three polymers were all around 100 000 Da, indicating similar reactivity of both monomers (Table S1 and Figure S1). Additionally, the PKL polypeptide with a K/L ratio of 2 exhibited only water solubility; however, when K/L ratios decreased to 1 and 0.5, the polymers became soluble in ethanol, which remarkably enhances their processability (Figure S2). Given that high hydrophobicity may compromise blood compatibility, we therefore selected PKL polypeptide with a K/L ratio of 1 as the polycation for the following studies.

To evaluate the electrostatic complexation capability, PAA and PKL were co‐dissolved in ethanol and then added dropwise into deionized water to form PKL‐PAA (KLA) polyplex suspension (Figure 1C). Transmission electron microscopy (TEM) and particle size analysis confirmed the formation of KLA nanoparticles in the solution, with an average diameter of approximately 153.7 nm (Figure 1D,E). As the molar ratio of positive charges from PKL to negative charges from PAA increased from 0.5 to 3, the zeta potential of the nanoparticles shifted from ‐32.5±1.1 to 31.8±2.3 mV (Figure 1F). After centrifugation and lyophilization, Fourier‐transform infrared (FTIR) spectroscopy was employed to analyze the composition of KLA nanoparticles (Figure 1G). Consistent with the zeta potential measurements, as the PKL/PAA molar ratio increased from 1 to 3, the carboxyl group peak in the polyplex dramatically decreased while the amide bond peak from PKL increased, indicating effective complexation. Compared to traditional methods for preparing polyelectrolyte complexes [41, 48], this KLA polyplex exhibits complete miscibility in ethanol and rapid formation of entangled complexes in the aqueous phase, offering a novel approach for the rapid construction of polyelectrolyte complex materials.

### 2.2 Antifouling and Cell Adhesion Behavior of KLA Coatings

**FIGURE 2:**
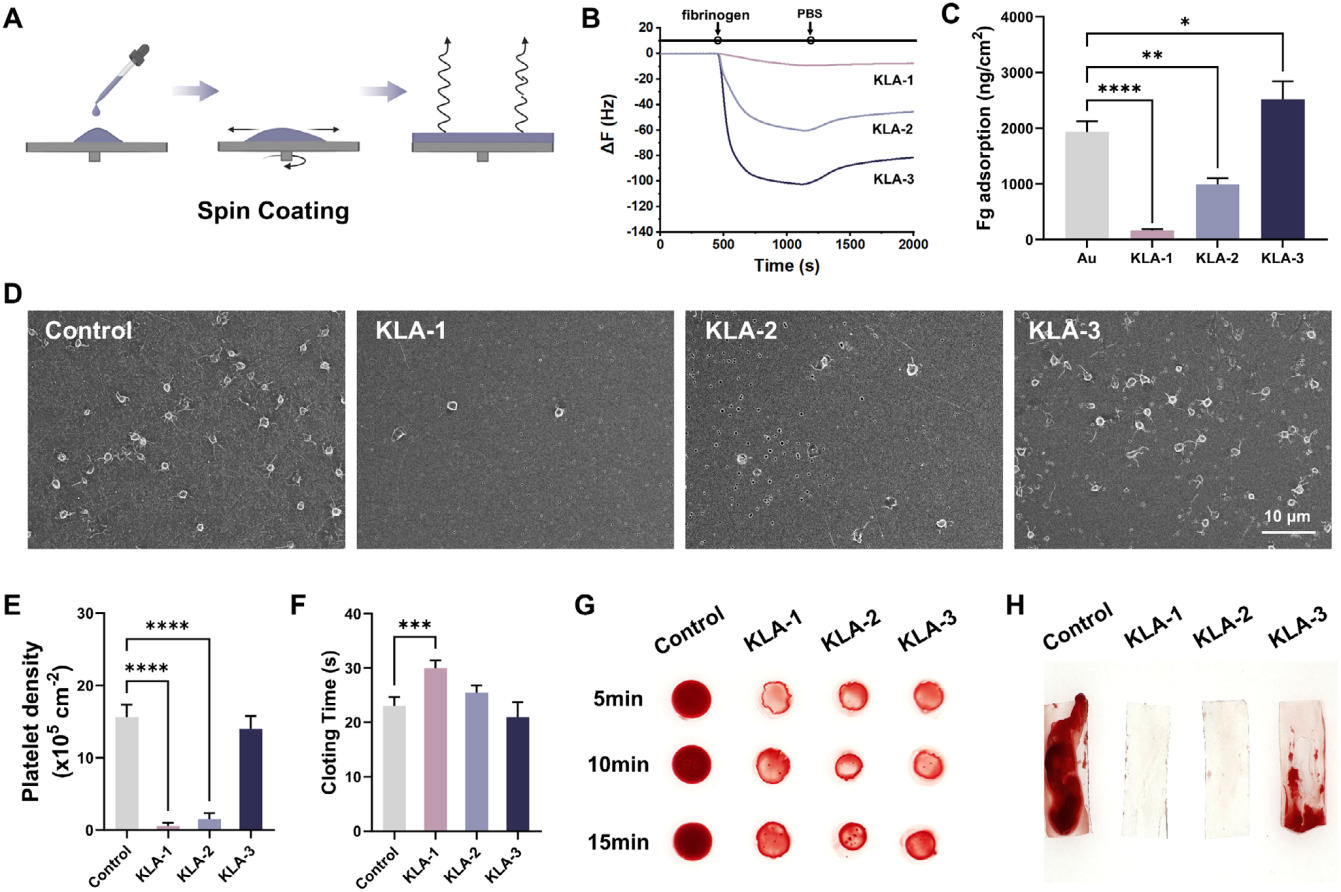
Antifouling properties of KLA coatings. (A) Schematic diagram of the preparation of KLA ultra‐thin coatings. Created with BioRender.com. The curves (B) and quantitative analysis (C) of Fg adsorption on gold chips and KLA coatings (*n* = 3). SEM micrographs (Scale bar: 10 µm) (D) and density (E) of platelets adhering to glass and KLA coatings (*n* = 3). APTT test (*n* = 3) (F), whole blood coagulation time test (G), and Chandler Loop model test (H) of substrates and KLA coatings. Data were presented as mean ± SD, and statistical significance was calculated by one‐way ANOVA with Tukey's multiple comparisons test (*p < 0.05; **p ≤ 0.01; ***p ≤ 0.001; ****p ≤ 0.0001).

We conducted a series of tests to evaluate the blood compatibility of KLA polyplexes. As illustrated in Figure 2A, KLA polyplexes were dissolved in ethanol and spin‐coated onto the surface of a gold chip to form an ultra‐thin coating. Compared with uncoated gold chips, the KLA‐3 samples, which exhibited the strongest positive charge, showed a more pronounced frequency shift, indicating a higher propensity for fibrinogen (Fg) adsorption (Figure 2B). As the proportion of PAA increased, the frequency shift of the KLA samples decreased significantly, demonstrating enhanced resistance to Fg adsorption. Quantitative protein adsorption analysis revealed that the adsorption of Fg on pure gold chips, KLA‐1, KLA‐2, and KLA‐3 was 1933 ± 193, 166 ± 23, 1424 ± 105, and 2517 ± 325 ng cm^−2^, respectively (Figure 2C). The platelet test results exhibited similar trends. Compared with the control glass substrate, the adsorption density of platelets on the KLA‐1 samples decreased by 98% (Figure 2D,E). Additionally, most platelets remained inactive, indicating that the negatively charged KLA‐1 samples significantly inhibited platelet adhesion and activation. Besides, the activated partial thromboplastin time (APTT) of the KLA‐1 samples reached 30.0 s, which was 33% longer than that of the uncoated glass substrates (Figure 2F). Moreover, the KLA‐1 samples exhibited excellent anti‐coagulation performance in whole blood experiments. As shown in Figure 2G, thrombus formation and adhesion were observed after static incubation of rabbit blood for varying durations. Fresh blood rapidly coagulated upon contact with the control glass substrate, leading to extensive thrombus adhesion on the surface within 15 min. In contrast, KLA‐1 samples exhibited a significant delay of blood coagulation, with a remarkable decrease in thrombus adhesion. These findings were further corroborated by dynamic whole blood tests using the Chandler loop (Figure 2H). The negatively charged KLA‐1 samples displayed superior resistance to fibrinogen adsorption, highlighting the potential for in vivo applications.

**FIGURE 3:**
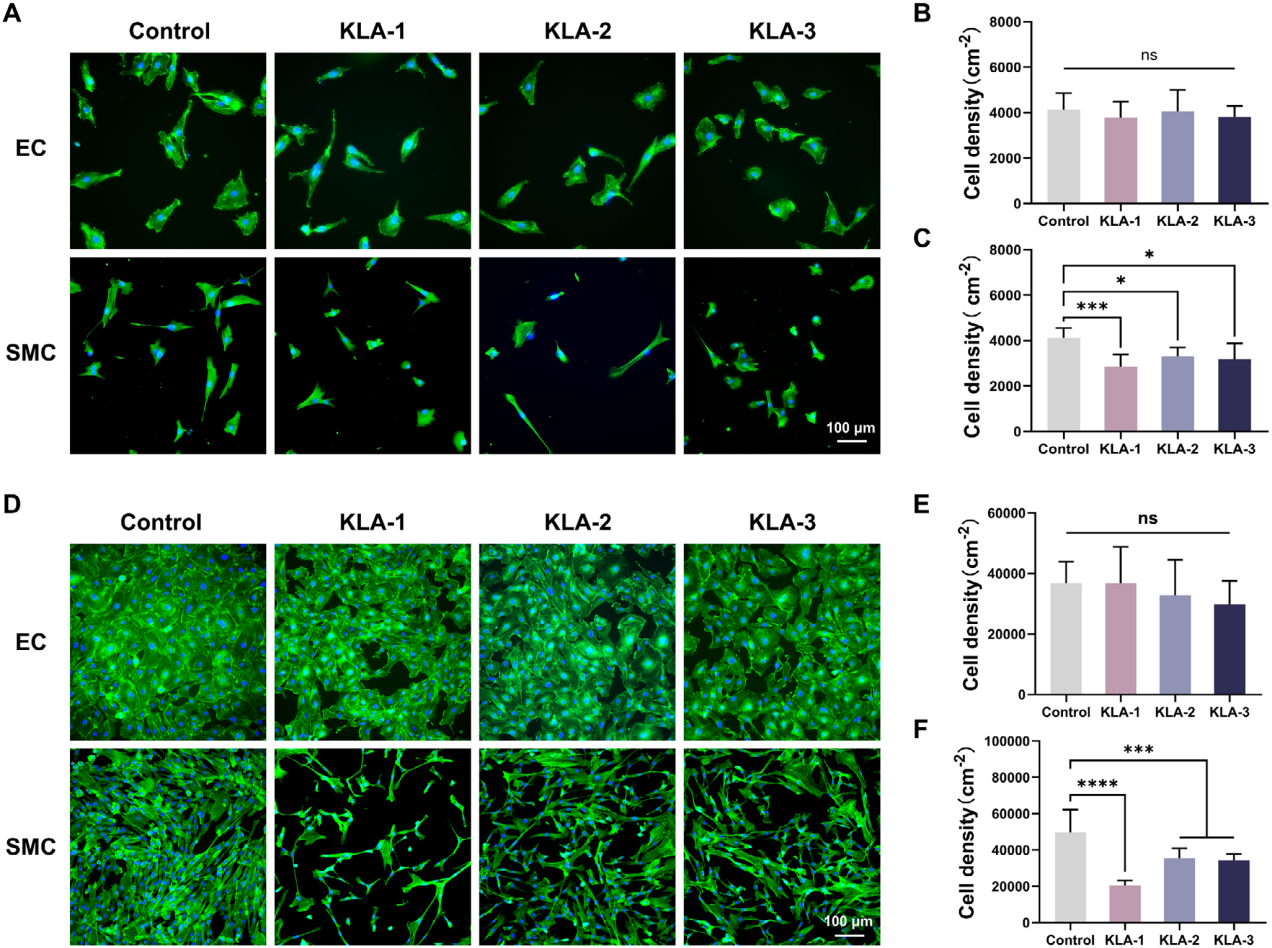
Cell adhesion and proliferation on KLA coatings. (A) Fluorescence micrographs of the adhesion of ECs and SMCs on TCPS and KLA coatings (blue: DAPI; green: F‐actin; Scale bar: 100 µm). Quantitative analysis of cell adhesion density of ECs (B) and SMCs (C) (*n* = 6). (D) Fluorescence micrographs of the proliferation of ECs and SMCs on TCPS and KLA coatings (blue: DAPI; green: F‐actin; Scale bar: 100 µm). Quantitative analysis of cell proliferation density of ECs (E) and SMCs (F) (*n* = 6). Data were presented as mean ± SD, and statistical significance was calculated by one‐way ANOVA with Tukey's multiple comparisons test (ns, not significant; ***p ≤ 0.001; ****p ≤ 0.0001).

Plenty of studies have demonstrated that effectively suppressing excessive proliferation of smooth muscle cells (SMCs) while maintaining the viability and functional integrity of endothelial cells (ECs) is crucial for endothelium regeneration following vascular interventions [49, 50, 51, 52, 53]. In this context, we investigated the impact of KLA polyplex composition on the adhesion and proliferation of ECs and SMCs. As shown in Figure 3A, ECs exhibited favorable morphology on the KLA coatings, with no significant difference compared to the TCPS group. Moreover, as the PKL ratio increased, the adhesion density of ECs showed an increasing trend. In contrast, the growth of SMCs on the KLA coatings was dramatically inhibited, with significantly lower adhesion density. Notably, the KLA‐1 samples exerted the most pronounced inhibition effect, reducing cell adhesion density by 58% (Figure 3C). In addition, the proliferation of ECs on the KLA coatings was comparable to that of the TCPS group, whereas the proliferation of SMCs was significantly inhibited. Specifically, the density of SMCs on the KLA‐1 samples decreased by 59% (Figure 3D–F). These results indicated that the KLA‐1 samples selectively inhibited the adhesion and proliferation of SMCs without affecting ECs. We further conducted co‐culture experiments to investigate the selective inhibitory effects of KLA coatings on SMCs (Figure S4). Fluorescence images revealed that, compared with the similar growth behavior on TCPS, the KLA‐1 samples dramatically suppressed the adhesion and proliferation of SMCs without interfering with the growth of ECs, which resulted in a notable EC/SMC density ratio of 2.5 after 72 h of co‐culture. Collectively, the superior anti‐coagulant performance and enhanced selective inhibition of SMCs indicated that KLA‐1 polyplex is a promising candidate for vascular regeneration.

To further investigate the selective inhibitory effect of the coating on SMCs, immunofluorescence staining was performed to examine cell adhesion morphology on both glass and coated surfaces. As shown in Figure S5, both ECs and SMCs spread well on the glass surface. On the KLA coatings, however, vinculin expression was markedly reduced in both cell types. Notably, SMCs exhibited significant contraction, whereas ECs extended pseudopodia. When cells were treated with an inhibitor of myosin II (blebbistatin), the morphological differences were altered: blebbistatin‐treated ECs adopted a starfish‐like shape, while SMCs displayed an irregular spread morphology. These observations suggest that intracellular contractile forces play a critical role in determining cellular adhesion behavior on the surface with fewer adhesion sites. Previous studies have demonstrated that, compared to ECs, SMCs exhibit higher phosphorylation of myosin II and generate stronger intracellular contractile forces [49, 54]. We therefore propose that the selective inhibition of SMC adhesion by the coating arises from intrinsic differences between the two cell types. Specifically, ECs possess randomly organized F‐actin and uniformly distributed adhesion sites, along with relatively low contractile forces, enabling effective spreading even on surfaces with limited adhesion sites. In contrast, SMCs exhibit highly aligned F‐actin and prominent vinculin localization predominantly at cell extremities, along with high contractile forces, which may impair stable adhesion and cell spreading.

### 2.3 UV‐Triggered Mechanical Enhancement of KLA Coatings

**FIGURE 4:**
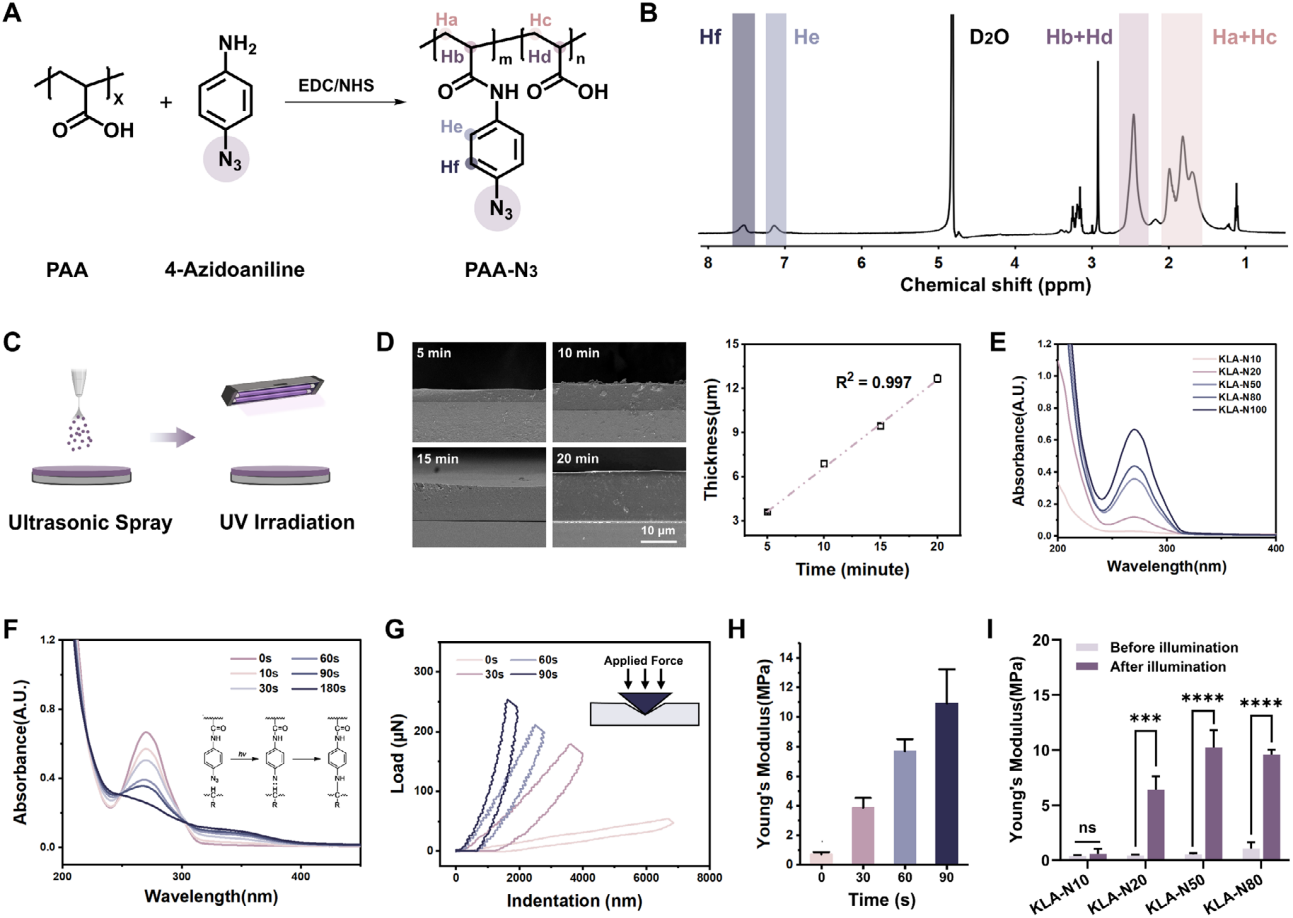
UV‐triggered mechanical enhancement of KLA coatings. (A) Schematic diagram of the synthesis of PAA‐N_3_. (B) ^1^H NMR spectrum of synthesized PAA‐N_3_. (C) Schematic diagram of the preparation of photo‐crosslinked KLA coating. Created with BioRender.com. (D) SEM micrographs and thickness trends of KLA coatings as spray time increases (*n* = 3; Scale bars: 10 µm). (E) UV–vis spectra of KLA coatings with different PAA‐N_3_ blending ratios before UV irradiation. (F) UV–vis spectra of KLA‐N20 coating before and after UV irradiation for different times. The Young's modulus (G) and curves (H) of KLA‐N20 samples before and after irradiation for various times (*n* = 3). (I) The Young's modulus of KLA coating with different PAA‐N_3_ blending ratios before and after irradiation (*n* = 3). Data were presented as mean ± SD, and statistical significance was calculated by one‐way ANOVA with Tukey's multiple comparisons test (ns, not significant; ***p ≤ 0.001; ****p ≤ 0.0001).

Previous studies have extensively adopted polyplexes to mimic the extracellular matrix for regulating cell behavior, attributed to their low mechanical strength and dynamic mechanical dissipation [55, 56, 57]. To enhance the mechanical strength, we introduced an azide functional group to PAA according to previous work (Figure 4A,B) [48], and developed mechanically reinforced KLA polyplex materials via UV‐induced crosslinking (Figure 4C). This ultrasonic spraying method allows precise control over coating thickness by adjusting the spraying duration (Figure 4D). As illustrated in Figure 4E, the characteristic absorption peak of the azide group at 270 nm increased with higher PAA‐N_3_ blending ratios. After 60 s of UV irradiation, the azide groups rapidly formed a covalent crosslinked network, thereby significantly enhancing the mechanical strength of the KLA coatings (Figure 2F–I). When the PAA‐N_3_ blending ratio increased to 20%, the Young's modulus of the KLA coatings in PBS reached 6.42 ± 0.97 MPa, approximately 15 times higher than the untreated coating (0.43 ± 0.07 MPa). As the azide group content increased to 50%, the modulus of the KLA coatings continued to improve, with the wet‐state Young's modulus reaching up to 10.3 ± 1.3 MPa. However, no further enhancement was observed when the blending ratio reached 80%. Moreover, the absorption peak of the azide group at 270 nm gradually diminished with UV irradiation, indicating that the crosslinking reaction can be controlled by adjusting the duration of UV exposure. Correspondingly, the Young's modulus of the coating increased with increasing irradiation time, ranging from 0.74 MPa to 10.9 MPa (Figure 4H). Consequently, the incorporation of PAA‐N_3_ in the KLA coating achieved a broad range of Young's modulus values, offering favorable mechanical flexibility for in vivo applications.

### 2.4 Drug Release Profile and Inhibition of SMCs Proliferation

**FIGURE 5:**
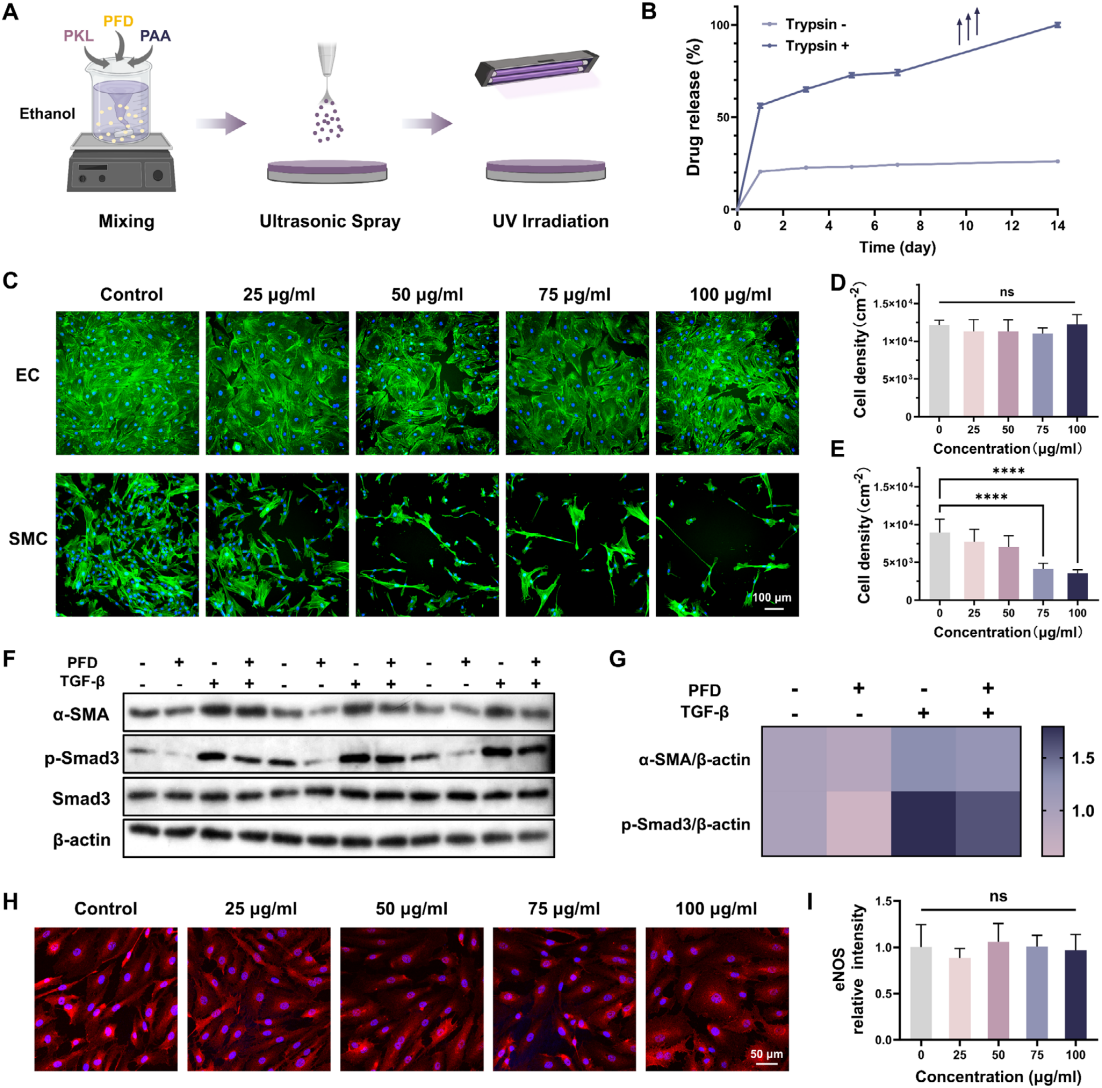
The preparation of PFD@KLA coatings and the drug release profile. (A) Schematic diagram of the preparation of PFD@KLA coating. Created with BioRender.com. (B) Drug release behavior of PFD@KLA coating (*n* = 3). Fluorescence micrographs of ECs and SMCs (blue: DAPI; green: F‐actin; Scale bar: 100 µm) (C), cell density of ECs (D) and SMCs (E) under different PFD concentrations (*n* = 6). Western blot analysis bands (F) and the relative expression (G) of proteins related to TGF‐β/Smad3 pathway (+ and ‐ represent the existence or absence of PFD and TGF‐β; PFD concentration: 100 µg mL^−1^; TGF‐β concentration: 10 ng mL^−1^; *n* = 6). Confocal images of ECs (blue: DAPI; red: eNOS; Scale bar: 50 µm) (H) and the relative intensity of eNOS expression (I) under different PFD concentrations (*n* = 6). Data were presented as mean ± SD, and statistical significance was calculated by one‐way ANOVA with Tukey's multiple comparisons test (ns, not significant; ****p ≤ 0.0001).

We further investigated the drug release behavior of the KLA coatings. Based on its solubility in ethanol, the KLA polyplex was mixed with the small molecule drug pirfenidone (PFD) and sprayed to prepare the PFD@KLA drug‐loaded coatings (Figure 5A). PFD was chosen as the model drug to evaluate the feasibility and therapeutic efficacy of in vivo intervention using the BEAT platform because of its significant inhibition of the proliferation of SMCs [58]. To simulate an in vivo environment, we evaluated the drug release behavior by immersing the drug‐loaded coating in a trypsin‐containing PBS solution. The PFD@KLA coating exhibited an initial burst release in the PBS solution, with 21% of the drug released within the first 24 h and 25% released over 14 d (Figure 5B). In contrast, in trypsin‐containing PBS solution, the release behavior was significantly accelerated, with 54% of the drug released within 24 h and 95% released over 14 d. Notably, after the seventh day in the trypsin environment, a secondary acceleration in drug release was observed, which may be attributed to the accelerated degradation of the coatings (Figure S9). Next, we evaluated the impact of pirfenidone on the proliferation of ECs and SMCs. As shown in Figure 5C, after a 48 h incubation at various concentrations, no significant difference was observed in EC density compared with the control group. In contrast, SMC density exhibited a remarkable reduction when the drug concentration exceeded 50 µg mL^−1^, with an elongated cell morphology. At a concentration of 100 µg mL^−1^, the SMC density decreased to 45% of the control group, indicating a substantial inhibitory effect (Figure 5E).

Furthermore, the Western blot (WB) analysis (Figure 5F,G) revealed that the expression levels of α‐SMA, COL‐1, and p‐Smad3/Smad3 in SMCs were significantly downregulated in the drug‐treated group compared to the control group. The addition of TGF‐β to the culture medium upregulated the expression of these proteins, while the further addition of pirfenidone exhibited a downward trend in p‐Smad3 expression again. This result indicated that the inhibitory effect of pirfenidone was associated with the suppression of the TGF‐β/Smad3 signaling pathway. Additionally, the fluorescence intensity of the eNOS signal (red) in the pirfenidone‐treated ECs exhibited no significant difference compared to the control group, suggesting that the pirfenidone treatment did not impair endothelial cell function (Figure 5H,I). In contrast to the non‐specific inhibitory effects of rapamycin, pirfenidone effectively inhibited SMC growth across a broad concentration range without compromising endothelial cell viability or function, which holds significant implications for vascular regeneration after injury.

### 2.5 The Transfer and Adhesion Behavior of BEAT and Ex Vivo Evaluation

**FIGURE 6:**
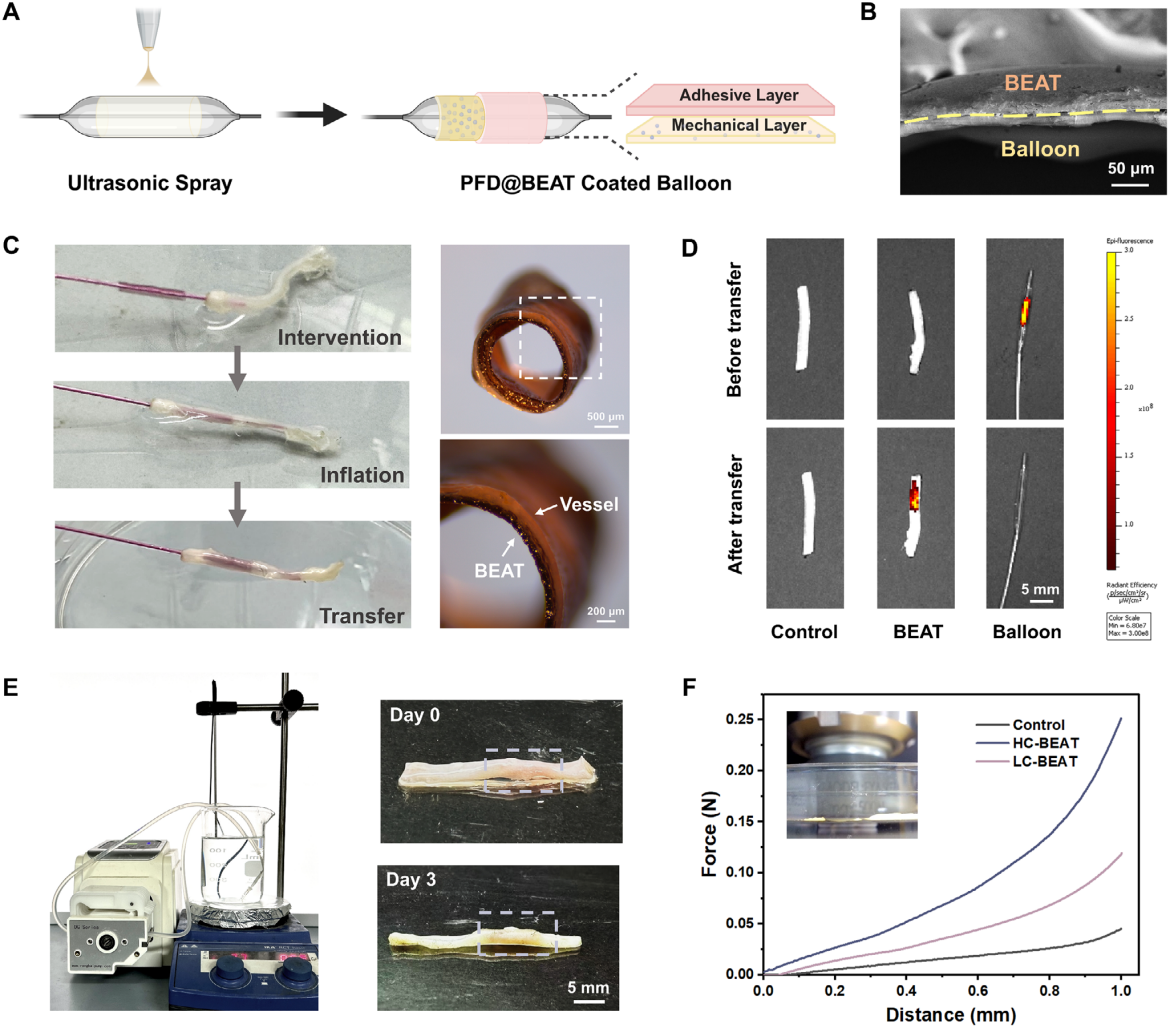
The preparation of PFD@BEAT coated balloon and ex vivo evaluation. (A) Schematic diagram of the preparation of PFD@BEAT coated balloon. Created with BioRender.com. (B) Cross‐sectional SEM micrographs of PFD@BEAT coated balloon (Scale bar: 50 µm). (C) Digital photos of ex vivo vascular intervention experiments, demonstrating that the BEAT coating can be successfully transferred to the blood vessel wall and achieve lumen patency (Scale bars: 500 and 200 µm). (D) Fluorescence images of PFD@BEAT coated balloons and rat abdominal aorta before and after transfer (Scale bar: 5 mm). (E) Digital photos of the blood vessel with PFD@BEAT coating before and after ex vivo circulation. (F) The compression curve of the blood vessel with and without BEAT coating under a wet environment (Scale bar: 5 mm).

To verify the endovascular transfer and mechanical enhancement, we employed 3D ultrasonic spraying technology to fabricate a BEAT coating on the balloon surface. This coating features a Janus multilayer structure, comprising a PFD@KLA mechanical layer and a poly(thioctic acid) (PTA) adhesion layer (Figure 6A,B). The adhesive PTA has been investigated for its ability to achieve robust adhesion on wet tissues, which can be attributed to the hydrophobic nature and abundant hydrogen bonding sites from carboxyl groups [45, 46, 59]. We further designed a polyvinylpyrrolidone (PVP) sacrificial layer on the balloon surface to ensure the rapid separation of the BEAT coating from the balloon substrate. After photo‐crosslinking, the PFD@BEAT coating demonstrated notable self‐supporting properties with reversible recovery of tubular structure after deformation (Figure S11 and Movie S1). As shown in Figure 6C, ex vivo vascular intervention experiments confirmed that the PFD@BEAT coating could be successfully transferred onto the vessel wall within 1 min of balloon inflation to achieve lumen patency. The fluorescence images further confirmed that the PFD@BEAT coating had been completely transferred to the vessel wall, with no residue remaining on the balloon surface (Figure 6D). We further established an ex vivo circulation model to simulate intravascular blood flow environment. As shown in Figure 6E, the PFD@BEAT coating remained intact within the blood vessel and provided effective vascular patency after 3 d of ex vivo circulation (Figure S12). To evaluate the mechanical enhancement provided by the BEAT coating on blood vessels, we conducted a compression test in a wet environment. Two types of BEAT coatings, low‐crosslinked BEAT (LC‐BEAT) and high‐crosslinked BEAT (HC‐BEAT), were prepared by UV irradiation for 30 s and 60 s, respectively. When applying 50% radial displacement of the original diameter (2 mm), the compression forces of the untreated vessel, LC‐BEAT‐treated vessel, and HC‐BEAT‐treated vessel were 0.045, 0.11, and 0.251 N, respectively. The LC‐BEAT coating demonstrated a significant 162% enhancement in radial force of the blood vessel, indicating superior potential to prevent elastic recoil compared with traditional drug‐eluting balloons.

**FIGURE 7:**
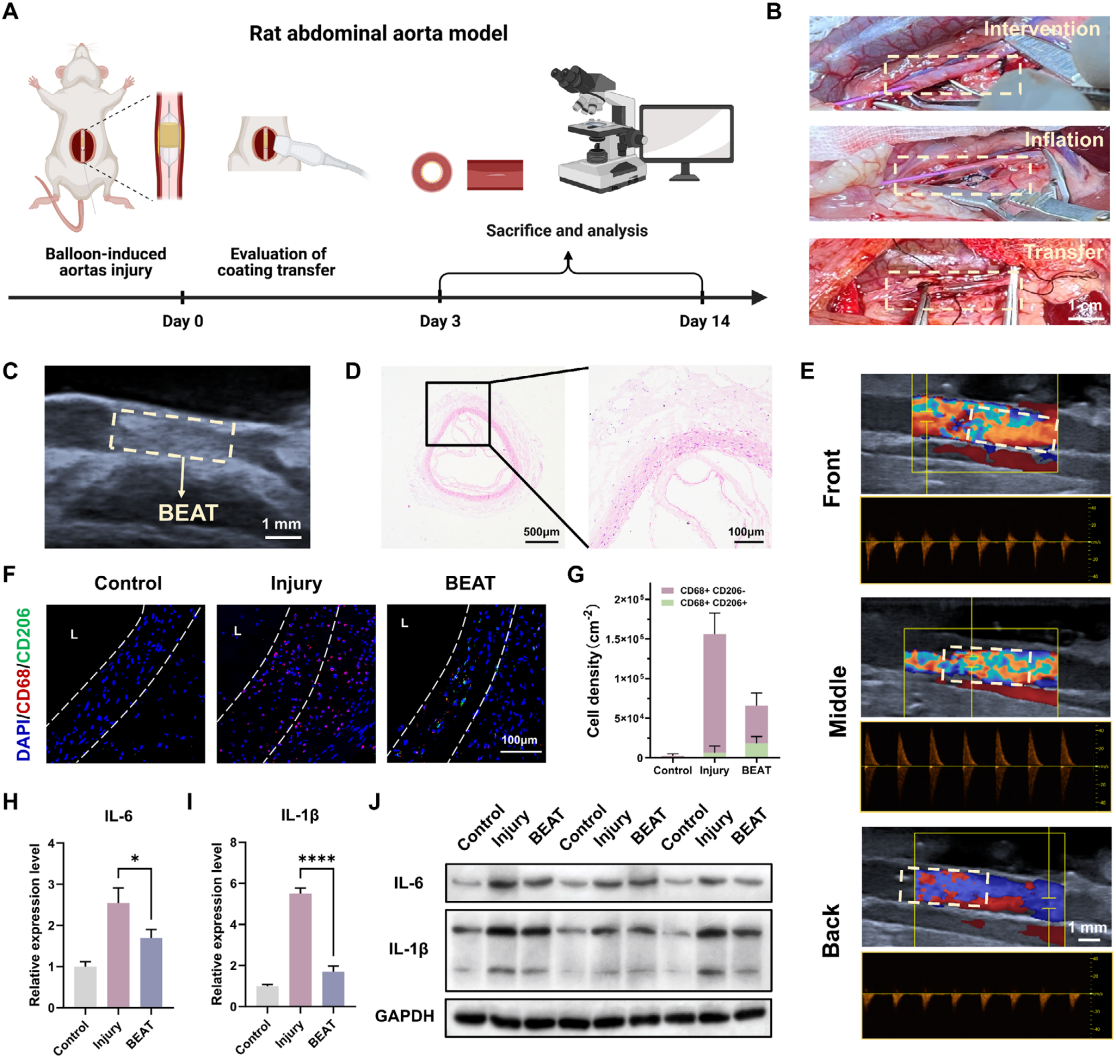
In vivo evaluation of PFD@BEAT coated balloon. (A) Schematic diagram of the rat abdominal aorta model. Created with BioRender.com. (B) Digital images of in vivo vascular intervention experiments (Scale bar: 1 cm). Doppler ultrasound images (Scale bar: 1 mm) (C) and H&E staining images (Scale bars: 500 and 100 µm) (D) of the blood vessel with BEAT coating immediately after implantation. (E) Doppler ultrasound images of the blood vessel with BEAT coating on day 3 (Scale bar: 1 mm). Representative double‐staining images for CD68 and CD206 (blue: DAPI; red: CD68; green: CD206; *n* = 3; Scale bar: 100 µm) (F), quantification of CD68^+^ CD206^−^ cells and CD68^+^ CD206^+^ cells (G), relative mRNA expression levels of IL‐6 (H) and IL‐1β (I) and Western blot analysis bands (J) of aortas from different groups on day 3 (*n* = 3). Data were presented as mean ± SD, and statistical significance was calculated by one‐way ANOVA with Tukey's multiple comparisons test (ns, not significant; *p ≤ 0.05; ****p ≤ 0.0001).

To test the applicability of the PFD@BEAT coating in balloon therapy, we established a rat abdominal aortic interventional model (Figure 7A). The PFD@BEAT coated balloon was introduced into the rat abdominal aorta. Upon inflation and expansion for 60 s, the PFD@BEAT coating rapidly detached from the balloon surface and adhered to the vessel wall (Figure 7B). Doppler ultrasound imaging confirmed (Figure 7C) that the PFD@BEAT coating was successfully transferred to the vessel wall with tight adhesion. Histological H&E staining (Figure 7D and Figure S13) further demonstrated efficient adhesion of the PFD@BEAT coating. No significant platelet adhesion and activation were observed on the PFD@BEAT coating within the blood vessels, highlighting the anti‐coagulation property of KLA polyplex. The partial detachment observed in histological sections was attributed to inconsistent contraction between the tissue and the coating during fixation and dehydration. Doppler ultrasound imaging was conducted at immediate implantation and 3 d thereafter. As shown in Figure 7E, the blood flow in all segments of the implantation site remained stable and unobstructed, with no thrombosis observed. Notably, we also observed that the PFD@BEAT coating exhibited mechanical compliance with the contraction and dilation of the blood vessels (Movie S2). In contrast, the PFD@HC‐BEAT coating resulted in severe thrombosis within one day after implantation, probably attributable to its high mechanical strength, which altered vascular hemodynamics and triggered thrombotic events (Figure S14).

Next, we evaluated the acute inflammatory response at the vascular injury site after 3 d of intervention. As shown in Figure 7F, the vascular intima of the injured group exhibited substantial CD68‐positive signals (red), indicating significant macrophage infiltration and a robust inflammatory response. In contrast, the number of CD68‐positive cells in the BEAT group was reduced by 58% compared to that in the injured group (Figure 7G). To further evaluate the inflammatory level, we performed qPCR (Figure 7H,I) and WB analysis (Figure 7J). Compared with the injured group, the mRNA expression levels of IL‐6 and IL‐1β in the BEAT group were reduced by 33% and 69%, respectively. Similarly, cytokine secretion levels also showed a downward trend for IL‐6 and IL‐1β. These results collectively demonstrated that the localized release of PFD significantly mitigated the inflammatory response induced by balloon intervention, thereby providing a favorable environment for subsequent vascular regeneration.

**FIGURE 8:**
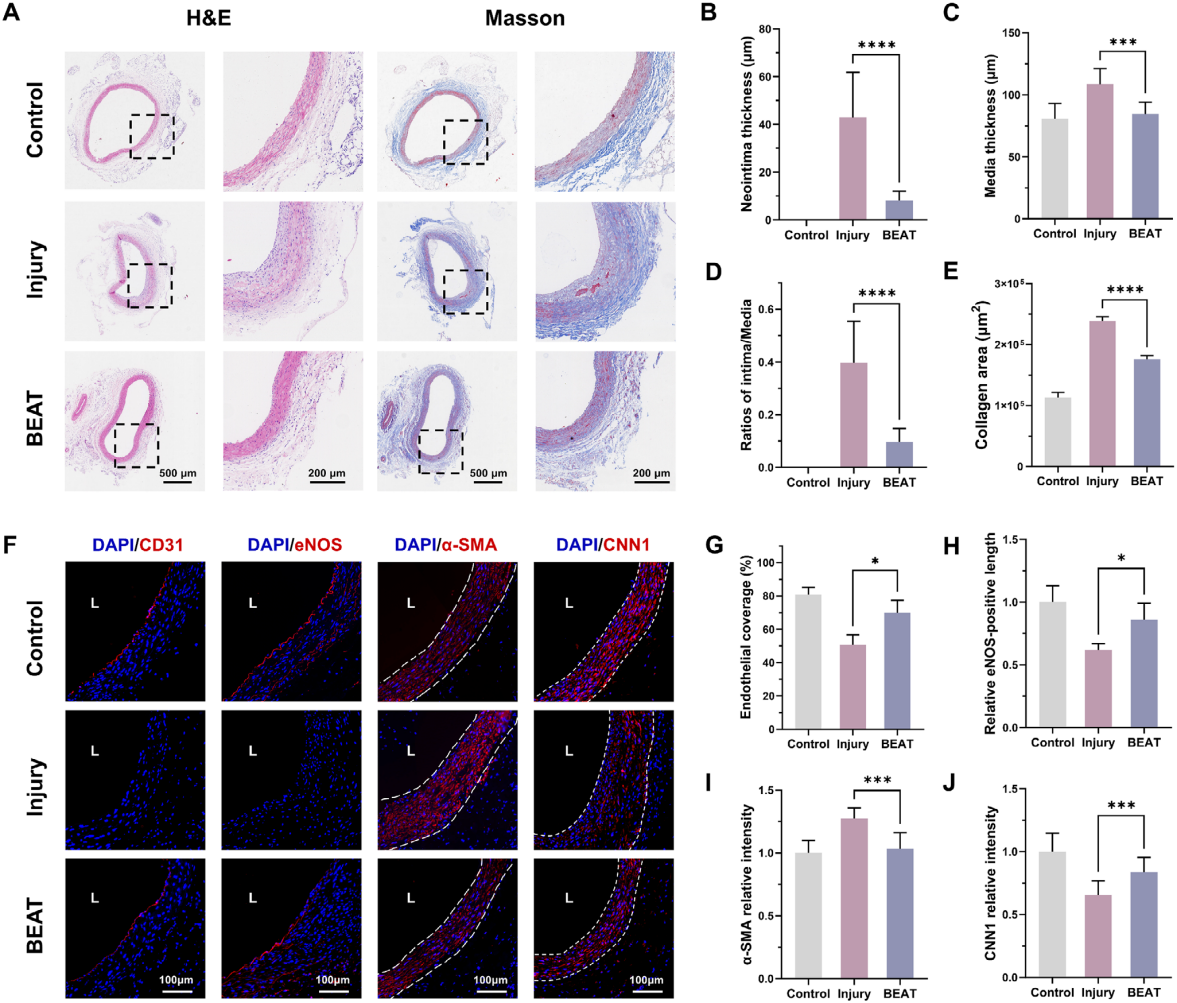
The vascular regeneration behavior of PFD@BEAT coating. (A) H&E and Masson's trichrome staining images of aortas from different groups on day 14 (Scale bars: 500 and 200 µm). Quantification of average neointima thickness (B), media thickness (C), ratios of intima/media (D), and collagen area (E) in different groups on day 14 (*n* = 3). (F) Representative immunofluorescent images for CD31, eNOS, α‐SMA, and CNN1 in different groups on day 14 (blue: DAPI; red: CD31, eNOS, α‐SMA, and CNN1; Scale bar: 100 µm). Quantification of endothelial coverage (G), relative eNOS‐positive length (H), and relative intensity of α‐SMA (I) and CNN1 (J) expression (*n* = 3). Data were presented as mean ± SD, and statistical significance was calculated by one‐way ANOVA with Tukey's multiple comparisons test (*p ≤ 0.05; ***p ≤ 0.001; ****p ≤ 0.0001).

To evaluate the vascular regeneration and endothelialization behavior after balloon intervention, we collected vascular tissue samples 14 d post‐implantation for histological analysis. As shown in Figure 8A, severe intimal hyperplasia and collagen deposition were observed in the injury group. In contrast, the BEAT group exhibited remarkable attenuation of intimal hyperplasia with no evident thrombosis or inflammatory response. Notably, the PFD@BEAT coating completely degraded within 14 d, which underscores the “leave nothing behind” feature of balloon therapy. Statistical analysis revealed that the neointima thickness in the BEAT group was reduced by 81% compared to the control group, indicating that the localized PFD release effectively inhibited neointimal hyperplasia (Figure 8B). Additionally, the media thickness (Figure 8C) and collagen fiber area (Figure 8E) were significantly reduced in the BEAT group. To further validate the selective inhibitory effect of the PFD@BEAT coating on SMC proliferation, we performed immunofluorescence staining for CD31, eNOS, α‐SMA, and CNN1. As shown in Figure 8F, CD31 expression was markedly decreased in the injury group (red), whereas it was completely restored in the BEAT group. Statistical analysis (Figure 8G) revealed that the endothelial coverage rate in the BEAT group reached 70%, representing a 38% increase relative to the injury group. Moreover, eNOS staining results (Figure 8H) showed a 39% improvement in the BEAT group relative to the injury group. These results indicate that the PFD@BEAT coating significantly promoted endothelial regeneration and functional recovery. For SMCs, α‐SMA expression (red) was decreased by 19% in the BEAT group relative to the injury group (Figure 8I), suggesting effective inhibition of SMC proliferation. Furthermore, the expression of CNN1 in the BEAT group was upregulated by 28% compared to the injury group, indicating that the PFD@BEAT coating substantially preserved the contractile phenotype of SMCs, thereby inhibiting SMC proliferation and intimal hyperplasia (Figure 8J). Taken together, this PFD@BEAT platform demonstrates superior absorbability, effectively inhibits intimal hyperplasia, and facilitates the rapid regeneration and functional restoration of the vascular endothelium, offering a promising approach for precision vascular interventional therapy.

## 3 Conclusion

In summary, we developed a bioabsorbable endovascular adhesive tape (BEAT) platform based on a hierarchical polyelectrolyte complex consisting of a crosslinked drug‐eluting PKL/PLA (KLA) layer and poly(thioctic acid) (PTA) adhesive layer. By precisely adjusting the PKL/PAA ratio, the KLA coating demonstrates remarkable anticoagulant properties, cytocompatibility, and a selective advantage of endothelial cells over smooth muscle cells. The UV‐triggered crosslinking of the KLA coating substantially enhances the mechanical properties, offering a wide tunable range from 0.74 to 10.9 MPa. The combination of the PTA outer layer endows this BEAT coating with rapid adhesion to the blood vessel wall under wet conditions, which realizes an efficient transfer of the BEAT coating after 60 s of balloon dilation and offers a significant 162% radial force enhancement of the blood vessel. More importantly, the in vivo intervention results confirm that the BEAT coatings not only maintain stable adhesion under continuous blood flow but also demonstrate excellent adaptability to vessel contraction. The BEAT‐coated balloon facilitates efficient delivery and localized release of pirfenidone, thereby alleviating inflammation caused by balloon injury and inhibiting neointimal hyperplasia postintervention. This BEAT platform holds enormous potential for enhancing the therapeutic efficacy of drug‐coated balloons and offers promising benefits in interventional therapy for other lumen systems.

## 4 Experimental Section

### 4.1 Materials

PAA (35 wt%, *M*w = 100 000), 4‐azidoaniline hydrochloride, polyvinylpyrrolidone (PVP, *M*w = 10 000), and IR‐780 iodide were purchased from Sigma‐Aldrich (Shanghai, China). Lithium hexamethyldisilazide (LiHMDS) was purchased from Energy Chemical (Shanghai, China). N6‐benzyloxycarbonyl‐L‐lysine‐N‐carboxyanhydride (Cbz‐L‐Lys NCA), L‐leucine‐N‐carboxyanhydride (L‐Leu NCA), trifluoroacetic acid (TFA), and DL‐thioctic acid (TA) were purchased from Macklin (Shanghai, China). 33% HBr solution in HAc and pirfenidone were purchased from J&K Scientific (Shanghai, China). Tetrahydrofuran (THF), petroleum ether, and ethanol were purchased from Sinopharm Chemical Reagent Co., Ltd (Shanghai, China). Phosphate‐buffered saline (PBS) was purchased from Sangon Biotech (Shanghai, China). The activated partial thromboplastin time (APTT) test kit was purchased from Shanghai Yuanye Bio‐Technology (Shanghai, China). Human umbilical vein endothelial cells (HUVECs, Catalog #8000, RRID: CVCL_2959), human umbilical artery smooth muscle cells (HUASMCs, Catalog #8030, RRID: N/A‌), endothelial cell medium (ECM), and smooth muscle cell medium (SMCM) were purchased from ScienCell (USA). ECM consists of 500 mL of basal medium, 25 mL of fetal bovine serum, 5 mL of endothelial cell growth supplement, and 5 mL of penicillin/streptomycin solution. SMCM consists of 500 mL of basal medium, 10 mL of fetal bovine serum, 5 mL of smooth muscle cell growth supplement, and 5 mL of penicillin/streptomycin solution. We confirm that all cell lines tested negative for mycoplasma contamination. CellTracker Green CMFDA Dye and CellTracker Red CMTPX Dye were purchased from ThermoFisher Scientific Co., Ltd (USA). Blebbistatin was purchased from Sigma‐Aldrich (Shanghai, China). Dulbecco's modified eagle medium (DMEM) was purchased from Gibco (USA). Fetal bovine serum (FBS) was purchased from Meilunbio (Dalian, China). Transforming growth factor‐beta (TGF‐β) was purchased from PeproTech (Shanghai, China). Fresh rabbit blood was supplied by the Laboratory Animal Research Center (Zhejiang University). Deionized water used in all experiments was obtained through a Milli‐Q water purification system.

### 4.2 Synthesis of Poly(L‐lysine)‐r‐poly(L‐leucine) (PKL)

The PKL was synthesized through LiHMDS‐initiated superfast ring‐opening polymerization of α‐amino acid N‐carboxyanhydrides (NCAs). Cbz‐L‐Lys NCA (0.92 g, 3.0 mmol) and L‐Leu NCA (0.94 g, 6.0 mmol) were dissolved in anhydrous THF (20 mL) in a round‐bottom flask under stirring. 1 mm solution of LiHMDS in THF (0.09 mL, 0.09 mmol) was rapidly added to the flask. The reaction proceeded at room temperature for 4 h under continuous stirring. The resulting mixture was precipitated by pouring it into petroleum ether. The precipitation was collected after centrifugation and dried under vacuum. The crude product was then dissolved in TFA (10 mL), followed by the dropwise addition of a 33% HBr solution in acetic acid (4 mL) while stirring in an ice bath to remove the benzyloxycarbonyl group. A white powder was obtained as the final product. The chemical structure of PKL was analyzed by ^1^H NMR (Bruker AVANCE III, 500 MHz). The molecular weight of PKL was analyzed by GPC using DMF supplemented with 0.01 m LiBr as the mobile phase (Waters 1515, USA).

### 4.3 Preparation of PKL/PAA (KLA) Polyelectrolyte Complex

The PKL and PAA were dissolved in ethanol at a concentration of 10 mg mL^−1,^ respectively, and then mixed under continuous stirring to form the KLA polyelectrolyte complex. The PKL/PAA volume ratio varied from 1.0 to 4.0. TEM (Hitachi HT7700, Japan) was used to observe the morphology of the KLA polyelectrolyte complex particles. The size and zeta potential of the particles were measured by Zetasizer Nano‐ZS (Malvern Instruments, UK). ATR‐FTIR (Nicolet 6700, Thermo Fisher Scientific, USA) was utilized to analyze the components of the KLA particles. The water contact angle tester (OSA 100, Germany) was used to characterize the hydrophobicity of the KLA coating.

### 4.4 Hemocompatibility Analysis

The QCM‐D measurements were conducted using a quartz crystal microbalance with dissipation monitoring (Q‐sense AB, Sweden). A clean quartz crystal was mounted in the QCM chamber, and the Fg solution (1 mg mL^−1^) was introduced at a flow rate of 100 µL min^−1^. Following this, PBS buffer was perfused into the chamber at the same flow rate to rinse the surface. The in situ frequency shifts over time were recorded.

For the platelet adhesion test, the samples were incubated with platelet‐rich plasma at 37°C for 2 h, followed by gently washing with PBS three times, and fixed with 4% glutaraldehyde for 30 min. After dehydration with a graded ethanol series (20%, 40%, 50%, 60%, 70%, 80%, 90%, 100%), all samples were observed by SEM (Hitachi S4800, Japan). For the APTT test, different samples were successively incubated with platelet‐poor plasma and APTT reagents (Yuanye Bioengineering, China). Plasma clotting time was recorded. Whole blood coagulation time (WBCT) was assessed at specified time points (5, 10, and 15 min). A Chandler loop model was utilized for dynamic blood incubation tests. Samples were placed in 6.4 mm diameter silicone tubes (Tygon ND‐100‐65) with a length of 35 cm. Each tube was filled with 5 mL of fresh blood and sealed to form a closed loop. The loops were rotated at 10 rpm at 37°C for 10 min. The samples were carefully taken out and gently washed with PBS. Macroscopic images of the samples were captured.

### 4.5 Cell Culture and Evaluation

For the adhesion test, ECs and SMCs were seeded onto different samples in 24‐well culture plates at a density of 5000 cells per cm^2^, respectively. After incubation for 12 h, all samples were fixed with 4% paraformaldehyde, stained with DAPI (1:100, Sigma) for nuclei and FITC‐labeled phalloidin (1:200, Sigma) for F‐actin, and then sealed with the antifade reagent. At least six fluorescence micrographs were captured for analysis (Ti2‐E, Nikon, Japan).

For the proliferation test, ECs and SMCs were seeded onto different samples in 24‐well culture plates at a density of 10,000 cells per cm^2^, respectively. After incubation for 48 h, all samples were fixed with 4% paraformaldehyde, stained with DAPI (1:100, Sigma) for nuclei and FITC‐labeled phalloidin (1:200, Sigma) for F‐actin, and then sealed with the antifade reagent. At least six fluorescence micrographs were captured for analysis (Ti2‐E, Nikon, Japan).

For the co‐culture on the KLA coatings, ECs and SMCs were stained with CellTracker Red CMTPX Dye and CellTracker Green CMFDA Dye, respectively, and then seeded on different samples at a density of 5,000 cells per cm^2^ (total 10,000 cells per cm^2^). After co‐culture in ECM, all samples were fixed with 4% paraformaldehyde, stained with DAPI (1:100, Sigma) for nuclei, and sealed with the antifade reagent. At least six fluorescence micrographs were captured for analysis (Ti2‐E, Nikon, Japan).

For adhesion morphology observation, ECs and SMCs were incubated on the different samples in a 24‐well culture at a density of 5,000 cells per cm^2^. After incubation for 4 h, all samples were fixed with 4% paraformaldehyde, stained with DAPI (1:100, Sigma) for nuclei and FITC‐labeled phalloidin (1:200, Sigma) for F‐actin, and anti‐Vinculin (1:100, Sigma) for Vinculin. A laser scanning confocal microscope (Zeiss LSM 900) was used to observe morphological features.

For the myosin inhibition test, ECs and SMCs were cultured in medium with 0.1 vol% blebbistatin for 7 d. Then, the cell adhesion test was carried out following the same approach as described above, using the myosin‐inhibited cells, while in this case, the medium was also supplemented with 0.1 vol% blebbistatin.

The experiment was replicated three times. The data presented are from a representative independent experiment that included six parallel samples. For each parallel sample, at least one fluorescence photograph was captured. Representative images from each group were selected for presentation.

### 4.6 Regulation of Azide Group

To prepare the PAA with different concentrations of azide groups, PAA and PAA‐N_3_ were separately dissolved in ethanol at a concentration of 10 mg mL^−1^, and then mixed in different ratios (20%, 50%, 80%, 100%). The PAA‐N_3_ was synthesized via an amidation reaction between the carboxyl groups of PAA and the amino groups of 4‐azidoaniline hydrochloride. The grafting ratio of PAA‐N_3_ was determined by analyzing the integrals of the three hydrogen atoms on the polymeric backbone and the four hydrogen atoms on the aromatic ring in the ^1^H NMR spectrum (Bruker AVANCE III, 500 MHz).

### 4.7 Preparation of KLA Coatings

The PAA ethanol solution with azide group (10 mg mL^−1^) was mixed with PKL ethanol solution (10 mg mL^−1^) under continuous stirring to form the KLA polyelectrolyte complex. The PKL/PAA volume ratio varied from 1.0 to 4.0. The ultrasonic spray machine (Ruidu Photoelectric Technology, Shanghai, China) was used to spray KLA coatings on the planes and balloon catheters (1.5 mm in diameter and 15 mm in length, Zylox‐Tonbridge, Hangzhou, China), and all coating processes were carried out at a flow rate of 20 µL min^−1^. The coating was then photo‐crosslinked by UV irradiation for 5 min (365 nm, 148.5 mW cm^−2^, Uvitron Intelli‐Ray 400, USA). The microstructure and thickness of the KLA coatings were characterized using scanning electron microscopy (SEM, Hitachi S4800 and SU8010, Japan). UV−vis spectrophotometer (UV‐8000, Shanghai Metash Instruments Co., Ltd, China) was employed to measure the absorbance spectra of KLA coating. The Young's modulus of the KLA coating was obtained from a nanoindenter (Piuma) with a 48.8 N m^−1^ probe. The indentation depth was maintained at 5 µm during testing, and the data were analyzed within a fitting curve range of 10% using Dataviewer software (Piuma). XPS (K‐Alpha, Thermo Scientific, USA) was used to analyze the composition of the KLA coating. To evaluate the swelling behavior of KLA coatings, circular samples with 20 mm diameter were immersed into PBS at 37°C. The samples were weighed at 1, 3, 7, and 14 d. The swelling ratio was calculated according to the following equation: $\mathrm{Swelling}\;\mathrm{ratio}=\frac{w_{t}-w_{0}}{w_{0}}$, *w*_0_ is the weight of the original coatings, and w_t_ is the weight of the swollen coatings at time point t. To evaluate the enzymatic degradation behavior of KLA coatings, samples were immersed in PBS solutions containing 0.25% trypsin at 37°C and weighed after 3, 7, and 14 d. SEM (Hitachi S4800, Japan) was used to observe the surface and cross‐sectional morphology of the coating.

### 4.8 Loading and Releasing of Pirfenidone

Pirfenidone, dissolved in ethanol at a concentration of 10 mg mL^−1^, was mixed with KLA at a volume ratio of 1:50. The PFD@KLA coating was prepared by ultrasonic spray on the PMMA substrate and photo‐crosslinked by UV irradiation for 5 min (365 nm, 150 mW cm^−2^, Uvitron Intelli‐Ray 400, USA). After being immersed in PBS and 0.25% trypsin‐containing PBS solutions for 1, 3, 5, 7, and 14 d, the content of pirfenidone in the release buffer was measured using UV–vis spectrophotometer (UV‐8000, Shanghai Metash Instruments Co., Ltd, China).

To evaluate the effect of pirfenidone, ECs and SMCs were seeded on glass coverslips at a density of 10 000 cells per cm^2,^ respectively, and treated with pirfenidone at different concentrations (25, 50, 75, 100 µg mL^−1^). After a 3‐day culture, all samples were gently washed with PBS and fixed with 4% paraformaldehyde. Cell staining was performed for nuclei (DAPI, 1:100, Sigma) and F‐actin (FITC‐labeled phalloidin, 1:200, Sigma). All samples were sealed with the antifade reagent. At least six fluorescence micrographs were captured for analysis (Ti2‐E, Nikon, Japan).

To investigate the signaling pathway of TGF‐β/Smad3, SMCs were seeded in six‐well culture plates at a density of 20 000 cells per cm^2^, and then the medium was replaced with DMEM containing 2% FBS. After 24 h of starvation, the cells were treated with TGF‐β (PeproTech 100–21C‐10UG) and pirfenidone for 24 h. The protein expression levels of collagen‐I (Col‐I, CST 72026), α‐smooth muscle actin (α‐SMA, Abcam ab7817), Smad3 (CST 9523), and phospho‐Smad3 (p‐Smad3, CST 9520) were evaluated by Western blot.

To evaluate the function of endothelial cells under the influence of pirfenidone, ECs were seeded on glass coverslips at a density of 20 000 cells per cm^2^ and treated with pirfenidone at different concentrations (25, 50, 75, 100 µg mL^−1^). After 24 h of culture, all samples were gently washed with PBS and fixed with 4% paraformaldehyde. Cell staining was performed for nuclei (DAPI, 1:100, Sigma) and endothelial nitric oxide synthase (eNOS, 1:1000, Sigma). All samples were sealed with the antifade reagent. At least 6 confocal fluorescence micrographs were taken for analysis (LSM900, ZEISS, Germany).

The experiment was replicated three times. The data presented are from a representative independent experiment that included six parallel samples. For each parallel sample, at least one fluorescence photograph was captured. Representative images from each group were selected for presentation.

### 4.9 Coating Transfer Ex Vivo

The PFD@BEAT coated balloon consisted of three layers. Firstly, a sacrificial layer of polyvinylpyrrolidone (PVP) (10 mg mL^−1^ in ethanol) was sprayed onto the balloon with a thickness of 10 µm. Secondly, KLA (10 mg mL^−1^ in ethanol) was sprayed onto the balloon as a tough layer with a thickness of 25 µm. Finally, PTA (10 mg mL^−1^ in ethanol) was sprayed onto the balloon as an adhesive layer with a thickness of 10 µm.

To evaluate the adhesive properties of the PTA layer, we prepared PTA coatings on the surface of the TPU substrate. For burst pressure test, a device consisting of a syringe pump, digital pressure gauge, and pressure chamber was built to measure the burst pressure. A 3.5 mm diameter hole was created on the artery. During the deionized water pumping process (2 mL min^−1^), the maximum pressure shown on the screen of the digital pressure gauge was recorded as the burst pressure. For lap‐shear tests (ASTM F2256), the adhesion area was fixed at 25 mm width × 10 mm length. All tests were conducted at a constant displacement rate of 5 mm min^−1^. The lap‐shear strength was calculated according to the following equation: $\mathrm{Shear}\;\mathrm{strength}=\frac{F_{L}}{w\times l}$. AFM was employed to measure the micro‐scale adhesion force of PTA. The PTA polymer chains were covalently grafted onto the probe surface, and the adhesion force between the modified probe and the hyaluronic acid substrate was quantitatively evaluated as the probe approached and retracted from the surface.

To visualize the coating, IR‐780 iodide was added to the KLA solution and co‐sprayed to label the KLA coating. The coated balloon was inserted into the abdominal aorta of male Sprague‐Dawley (SD) rats, inflated with 3 atm pressure, and kept at this pressure for 2 min. Subsequently, the balloon was deflated and withdrawn from the abdominal aorta. The treated abdominal aorta was imaged using an IVIS Spectrum system (PerkinElmer, USA). Control images of the normal abdominal aorta and the coated balloons were also captured before and after the ex vivo application. An ex vivo circulation model was further established to simulate the blood flow environment within the vessels. Both ends of the blood vessels were connected to tubes, and a peristaltic pump circulated PBS solution at 37°C through the tubes at a flow rate of 20 cm s^−1^. Compression tests were conducted in a wet environment to evaluate the mechanical enhancement of the BEAT coating on blood vessels. Blood vessels were soaked in PBS and subjected to compression at a rate of 1 mm min^−1^ while recording the corresponding stress values.

### 4.10 Evaluation of In Vivo Transfer and Treatment

The animal use protocol listed below has been reviewed and approved by the Institutional Animal Care and Use Committee (IACUC, ZJCLA) (Approval No.ZJCLA‐IACUC‐20020191). Male Sprague‐Dawley (SD) rats (8–10 weeks in age, 350–400 g in weight) were used to evaluate in vivo transfer and treatment efficacy. To create abdominal aorta balloon‐induced vascular injury models, an abdominal midline incision was made, and the abdominal aorta was carefully exposed. A bare balloon catheter was inserted into the abdominal aorta and inflated to a pressure of 3 atm. The inflated balloon was subsequently withdrawn and advanced five times to induce vessel injury. Following this, the PFD@BEAT coated balloon was inserted into the injured vessels, inflated with 3 atm pressure, and maintained at this pressure for 2 min to facilitate drug transfer. After deflation and removal of the balloon, the surgical site was closed with sutures. During the procedure, the rats were fully anesthetized using chloral hydrate. Following deep anesthesia induced by an appropriate dosage of the agent, the animals were euthanized in a humane manner in accordance with ethical guidelines.

Ultrasound with color Doppler (VINNO D6VET, VINNO) was performed to evaluate blood flow at predetermined time points (immediately and 3 d post‐procedure). Three days after the procedure, half of the rats were anesthetized, and the abdominal aortas were harvested for H&E staining, immunostaining (CD68, CD206), Western blot analysis (IL‐6, IL‐1β), and quantitative real‐time PCR (qPCR) (IL‐6, IL‐1β) to assess the inflammatory response. On day 14, the other abdominal aortas were harvested from the anesthetized rats for H&E staining, Masson's trichrome staining, and immunostaining (CD31, eNOS, α‐SMA, CNN1) to further evaluate the level of endothelialization. Abdominal aortas from healthy rats without implantation served as controls. All stained samples were imaged with a fluorescence microscope (BX63, Olympus, Japan). For quantitative analysis of fluorescence intensity, we selected regions of equal area on each slice, measured the fluorescence intensity using ImageJ, and normalized the values by the corresponding cell count. The relative fluorescence intensity was then calculated following normalization to enable comparison among groups. All antibodies employed in immunofluorescence were derived from rabbits. The following primary antibodies were used: CD68 (1:300), CD206 (1:1000), CD31 (1:4000), eNOS (1:50), α‐SMA (1:300), and CNN1 (1:1000). Nuclei were counterstained with DAPI (1:10000).

In the evaluation of in vivo transfer and treatment, 3 rats were used for each group. Specifically, three blood vessels were separately collected from three individual rats. For tissue sections, 2–3 tissue slices were prepared from each rat, and one representative image was chosen from each blood vessel for statistical analysis.

### 4.11 Statistical Analysis

All statistical analyses were performed using GraphPad Prism (Version 10.1.2). Data are presented as mean ± standard deviation (SD). To ensure reproducibility of results, each experiment was repeated at least three times. For comparisons between two groups, a two‐tailed Student's t test was used. For comparisons among multiple groups, one‐way ANOVA with Tukey's multiple comparisons test was applied. A *p* value of less than 0.05 was considered statistically significant (*p < 0.05, **p ≤ 0.01, ***p ≤ 0.001, and ****p ≤ 0.0001).

## Conflicts of Interest

The authors declare no conflicts of interest.

## Supporting information




**Supporting File 1**: advs74296‐sup‐0001‐SuppMat.docx.

**Supporting File 2**: advs74296‐sup‐0002‐MovieS1.mp4.

**Supporting File 3**: advs74296‐sup‐0003‐MovieS2.mp4.

## Data Availability

The data that support the findings of this study are available from the corresponding author upon reasonable request.
