# Supplementary material for: Bioabsorbable Endovascular Adhesive Tape (BEAT) for Improving Vascular Regeneration
Source: Adv Sci (Weinh). 2026 Feb 10;13(22):e12857. doi: 10.1002/advs.202512857 (PMC13088294; doi:10.1002/advs.202512857)
Supplement: Supplementary file 1 — Supporting File 1: advs74296‐sup‐0001‐SuppMat.docx. [file ADVS-13-e12857-s001.docx]

Supporting Information

Bioabsorbable Endovascular Adhesive Tape (BEAT) for Improving Vascular Regeneration

Jiarong Wang^1,2^, Jing Wang^1,3^*, Xinyi Li^1,2^, Bo Yu^1,2^, Yiduo Chen^1,2^, Yirong Guo^1,2^, Honglin Qian^1,3^, Meng Hu^1,2^, Haoyang Liu^1,2^, Wenhui Liu^1,2^, Han Xu^2^, Kefeng Ren^1,2,3^, M. Cristina L. Martins^4^, Jian Ji^1,2,3^*

*^1^ State Key Laboratory of Transvascular Implantation Devices, Department of Cardiology, The Second Affiliated Hospital, School of Medicine, Zhejiang University, Hangzhou, P.R. China;*

*^2^ MOE Key Laboratory of Macromolecular Synthesis and Functionalization, Department of Polymer Science and Engineering, Zhejiang University, Hangzhou, P.R. China;*

*^3^ Transvascular Implantation Devices Research Institute, Hangzhou, P.R. China;*

*^4^ I3S-Instituto de Investigaçāo e Inovaçāo Em Saúde, Universidade Do Porto, INEB-Instituto de Engenharia Biomédica, Portugal;*

*These authors contributed equally: Jiarong Wang, Jing Wang*

**Corresponding author. E-mail: wangjing2015@zju.edu.cn; cmartins@i3s.up.pt; jijian@zju.edu.cn*

| **Sample** | **K/L ratio** | **Mn** | **Mw** | **DPI** |
| --- | --- | --- | --- | --- |
| PKL-1 | 1/1 | 1.02×10^5^ | 1.41×10^5^ | 1.39 |
| PKL-2 | 2/1 | 8.05×10^4^ | 1.02×10^5^ | 1.26 |
| PKL-3 | 1/2 | 1.07×10^5^ | 1.80×10^5^ | 1.69 |

**Table S1.** The molecular weight and molecular weight distribution of PKL.

| **Viscosities (cP)** | **PVP** | **KLA** | **PTA** |
| --- | --- | --- | --- |
| Test 1 | 26.36 | 17.80 | 22.62 |
| Test 2 | 26.76 | 18.10 | 22.71 |
| Test 3 | 26.75 | 18.89 | 22.45 |
| **Average** | **26.62** | **18.26** | **22.59** |

**Table S2.** The viscosities of the solutions for ultrasonic spray.


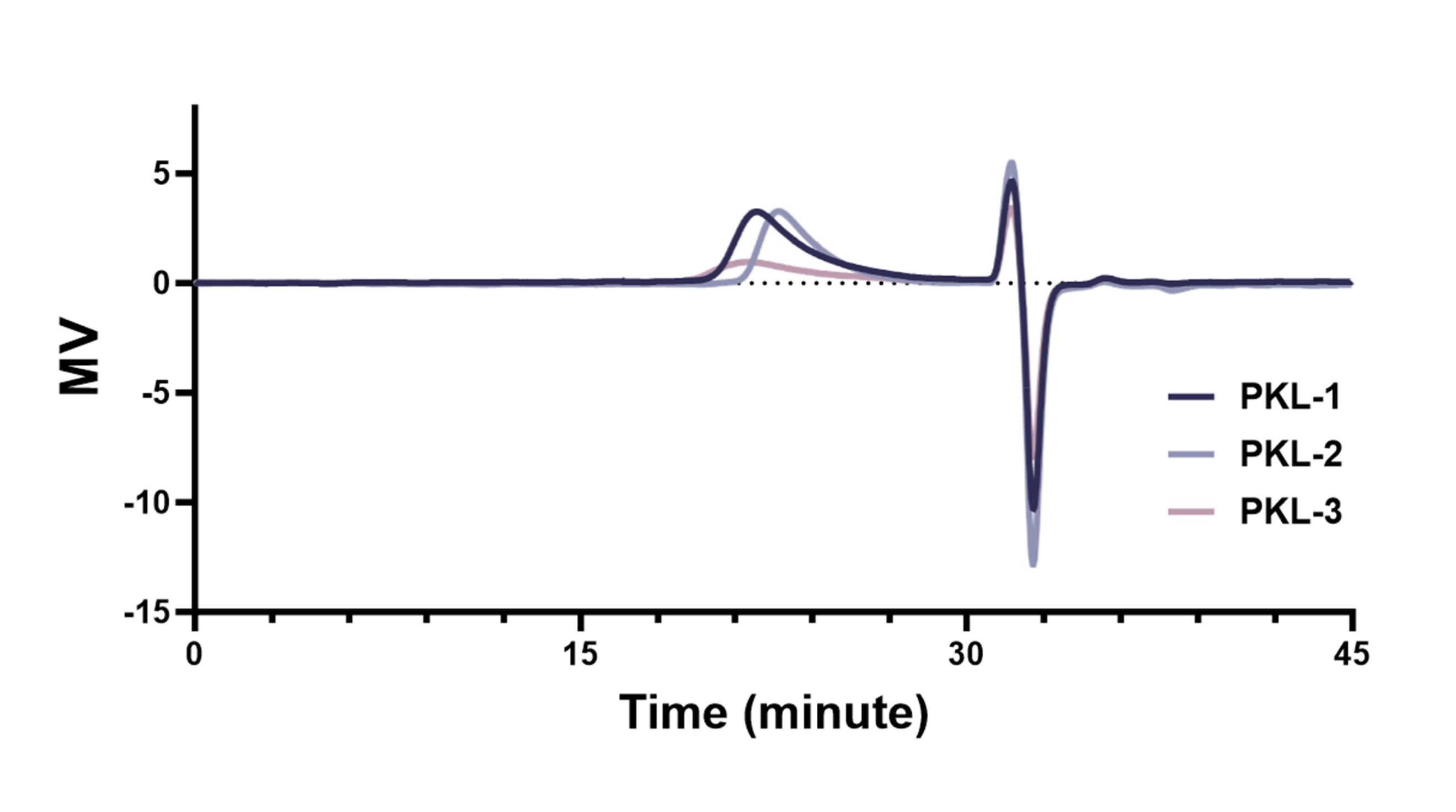


**Figure S1.** The GPC curve of PKL.


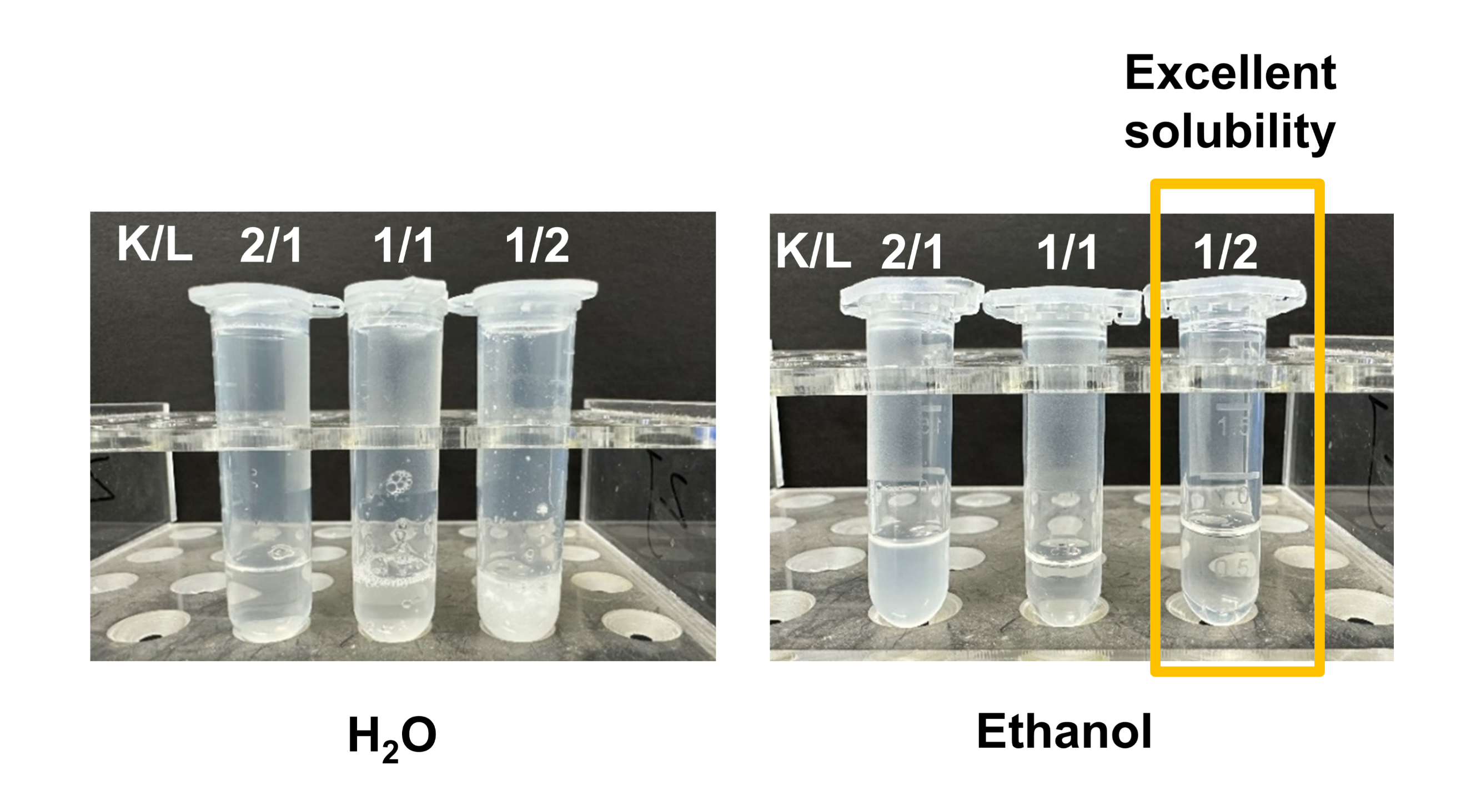


**Figure S2.** The Solubility of PKL in Water and Ethanol.


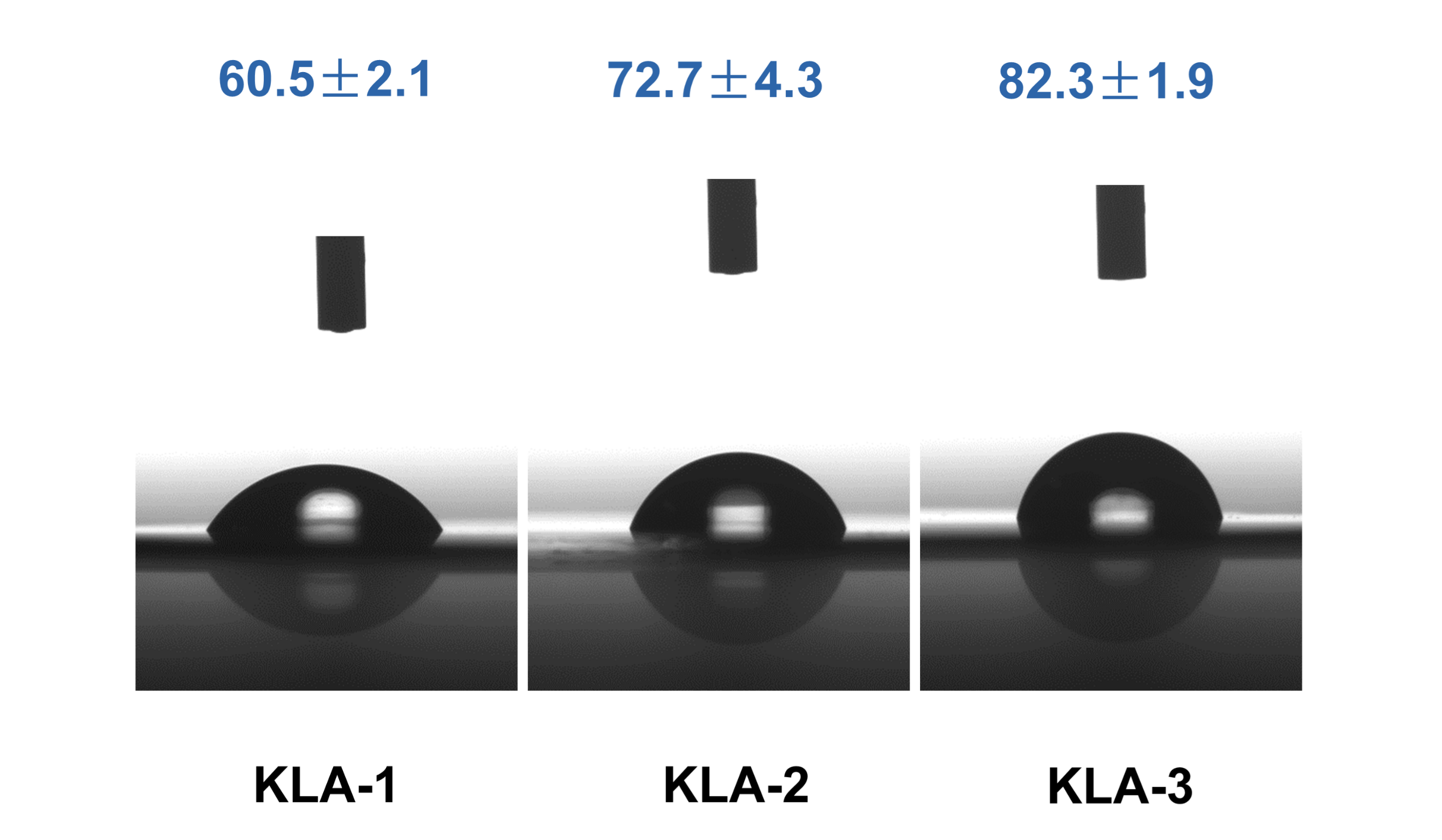


**Figure S3.** The Water contact angle of KLA coatings (n = 3). Data were presented as mean ± SD.


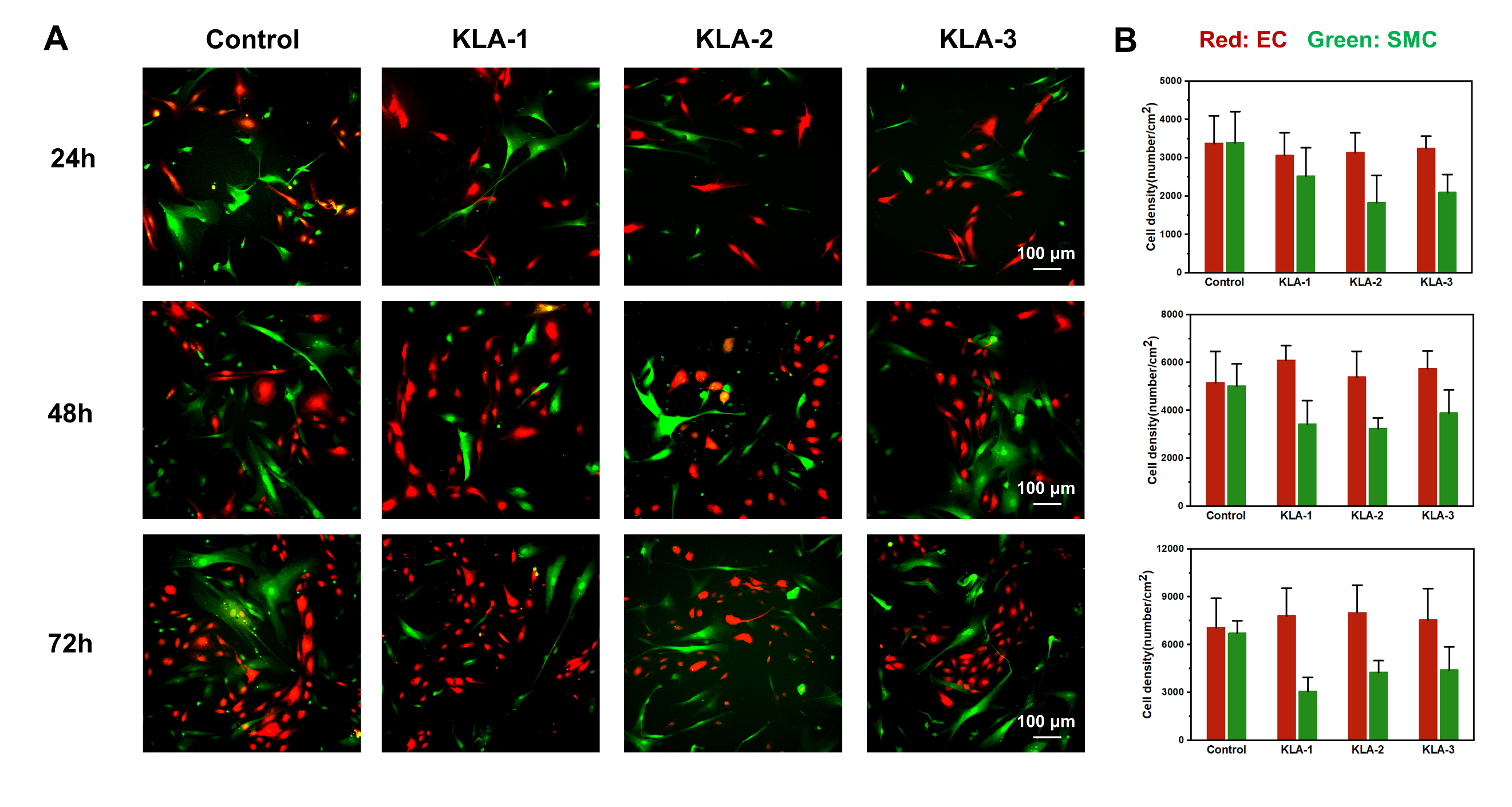


**Figure S4.** Co-culture of ECs and SMCs on KLA coatings. (A) Fluorescence micrographs of co-culture cells on different samples (red: EC; green: SMC; Scale bar: 100 μm). (B) Cell density of ECs and SMCs (n = 6). Data were presented as mean ± SD.


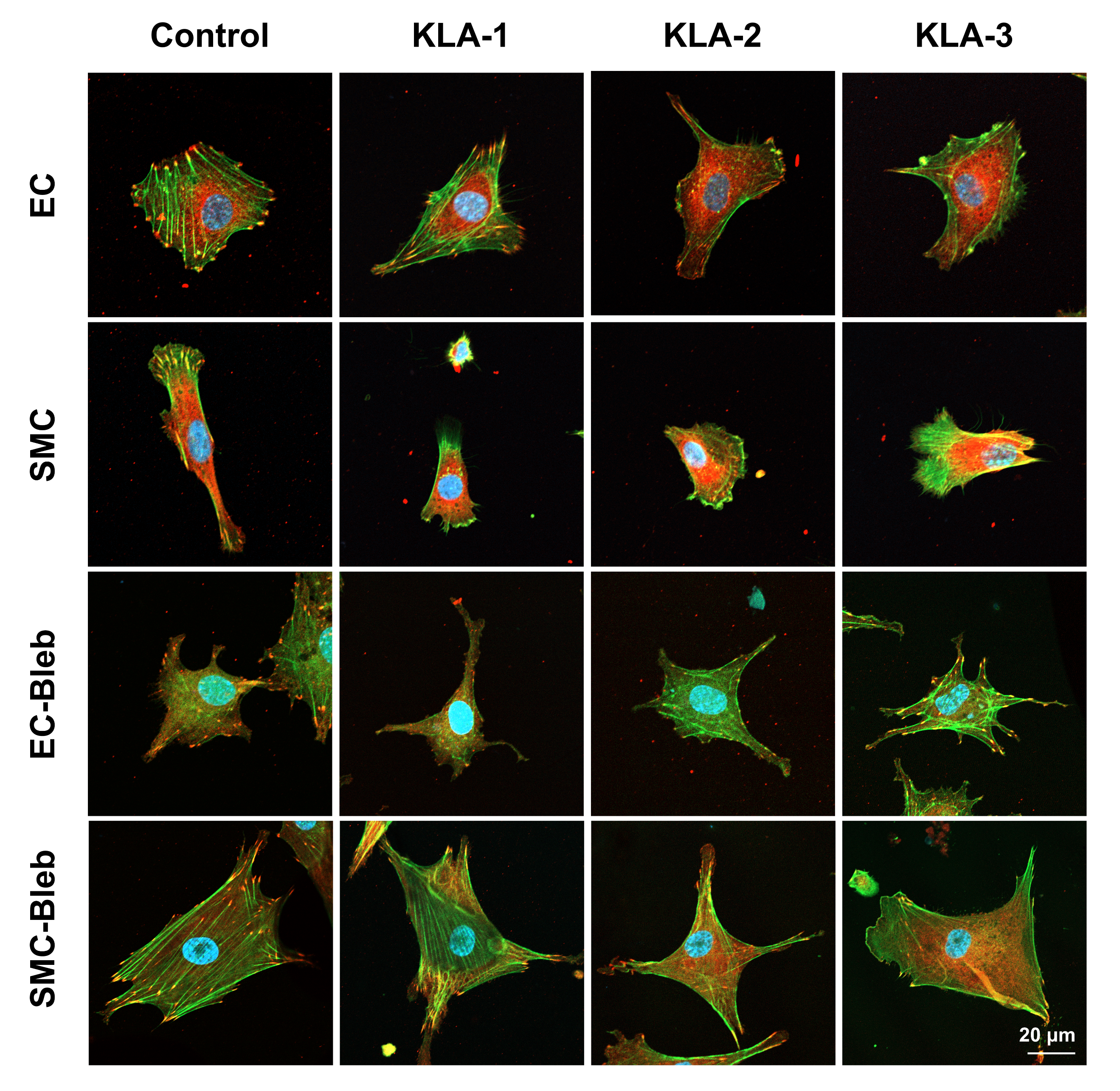


Figure S5. The confocal microscopy images of vinculin (red), F-actin (green), and nuclei (blue) immunofluorescence of ECs and SMCs with or without treatment of blebbistatin on KLA coatings (Scale bar: 20 μm).


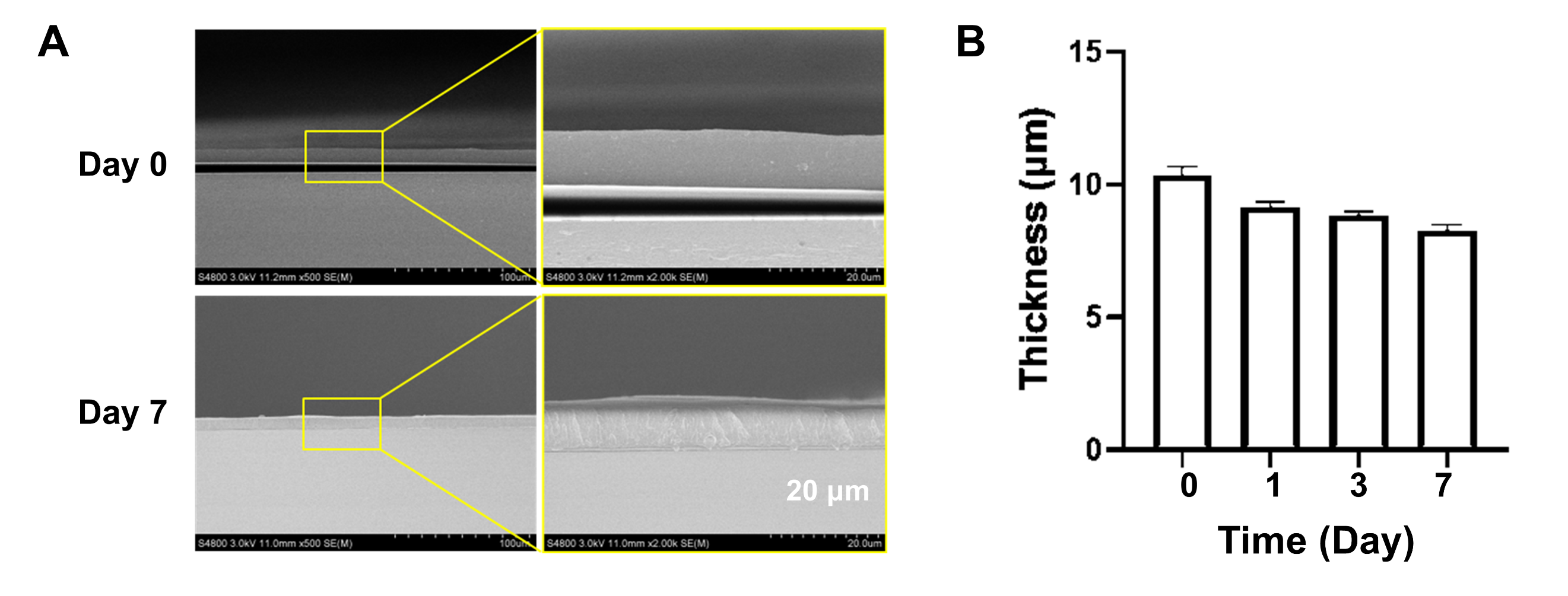


**Figure S6.** The stability of KLA coatings in PBS (n = 3; Scale bar: 20 μm). Data were presented as mean ± SD.


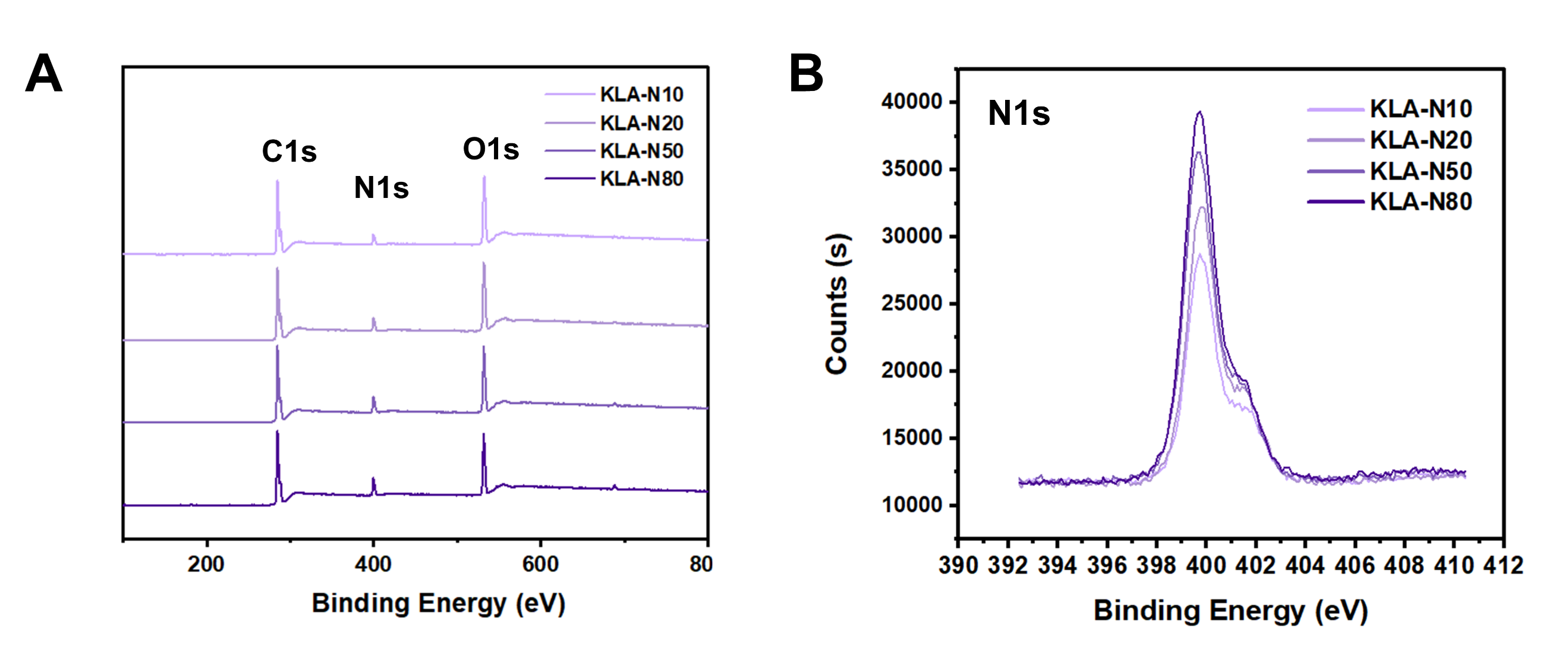


**Figure S7.** XPS analysis of KLA coatings with different PAA-N_3_ blending ratios.


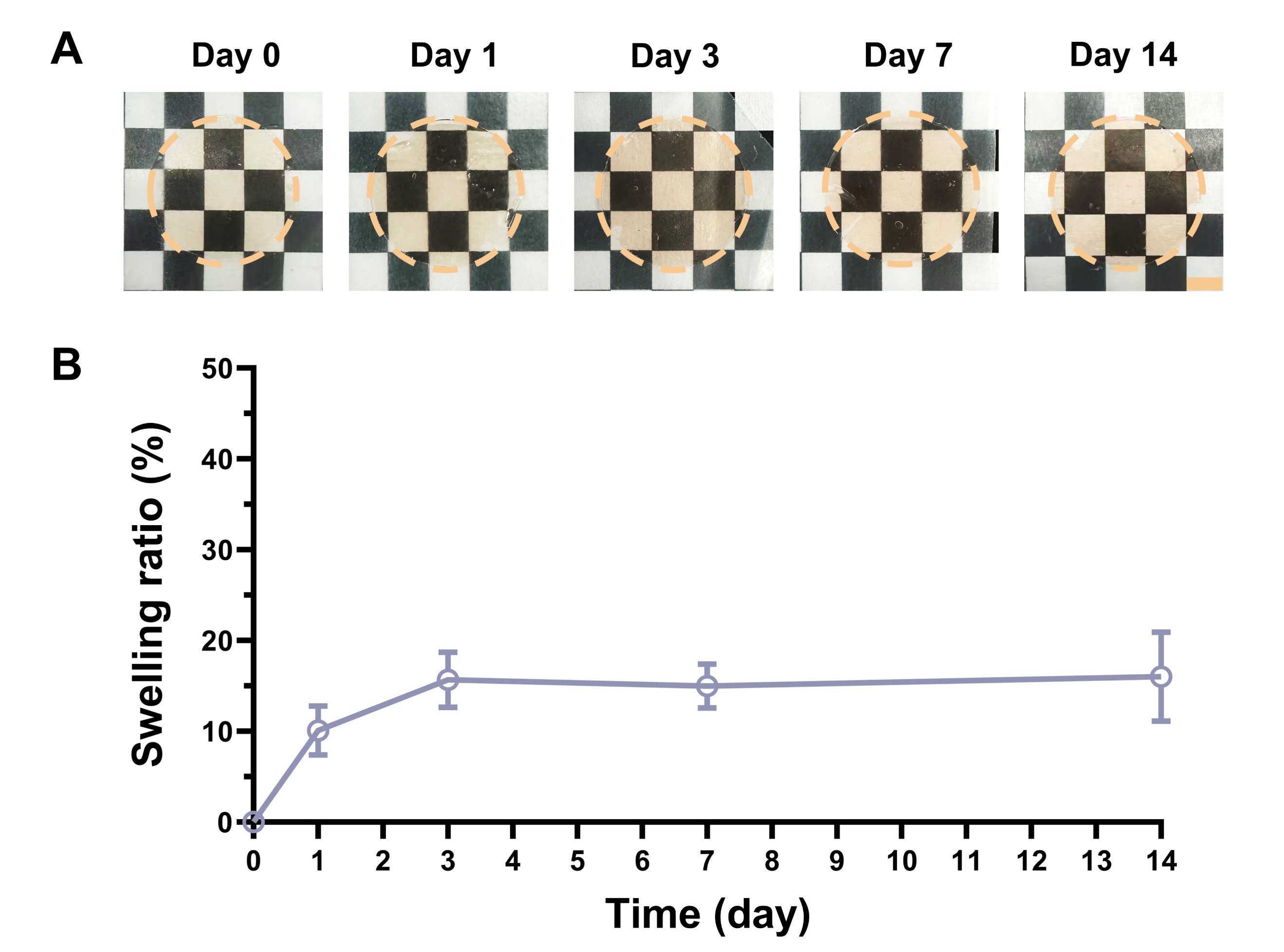


**Figure S8.** Anti-swelling performance of KLA coatings. (A) Digital photos (Scale bar: 5 mm) and (B) quantitative analysis of swelling ratios after immersion in PBS buffer for 1, 3, 7, and 14 days (n = 3). Data were presented as mean ± SD.


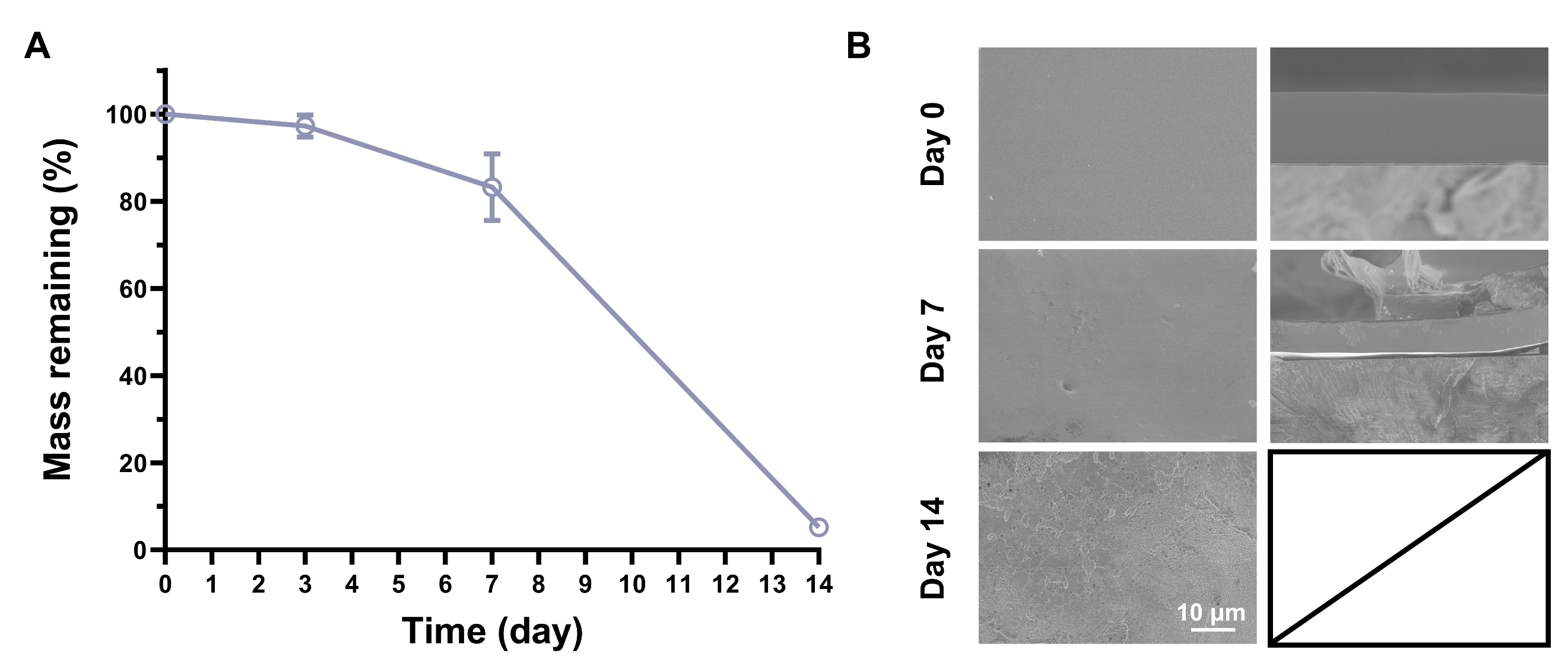


**Figure S9.** The enzyme-accelerated degradation behavior of KLA coatings. (A) Degradation curve of KLA coatings (n = 3). (B) SEM images of KLA coating following 7 and 14 days of degradation (Scale bar: 10 μm). Data were presented as mean ± SD.


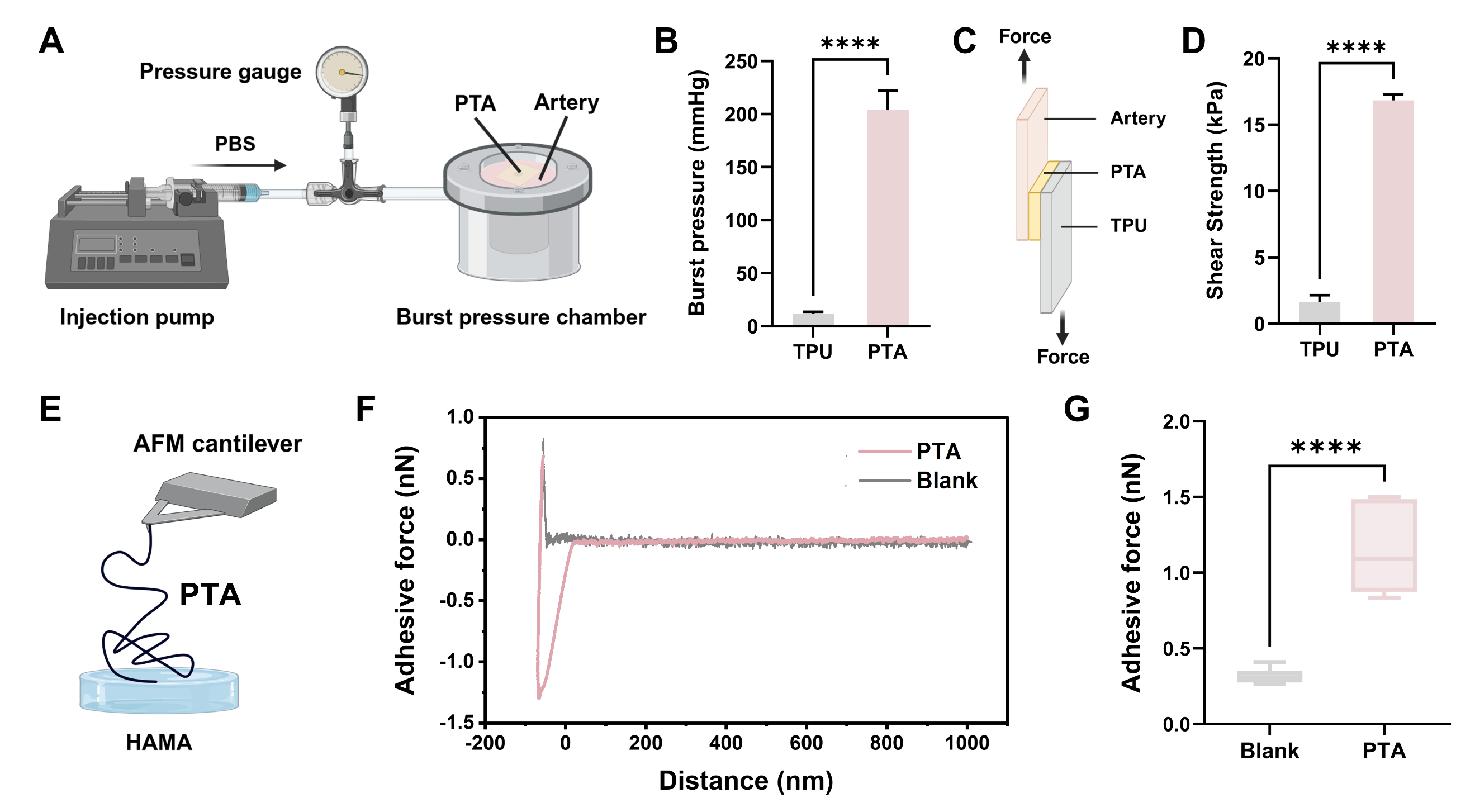


**Figure S10.** Tissue adhesive performance of the PTA layer. (A) Scheme of burst pressure test. Created with BioRender.com. (B) Burst pressures of the PTA layer and TPU substrate on the endothelium layer of the bovine artery (n = 3). (C) Scheme of 180-degree lap shear test. (D) Lap shear strength of the PTA layer and TPU substrate to the endothelium layer of the bovine artery (n = 3). (E) Scheme of microscopic adhesion force test. (F) Force-displacement curves and (G) Microscopic adhesion force of PTA (n = 3). Data were presented as mean ± SD, and statistical significance was calculated by two-tailed Student's t test (****p ≤ 0.0001).


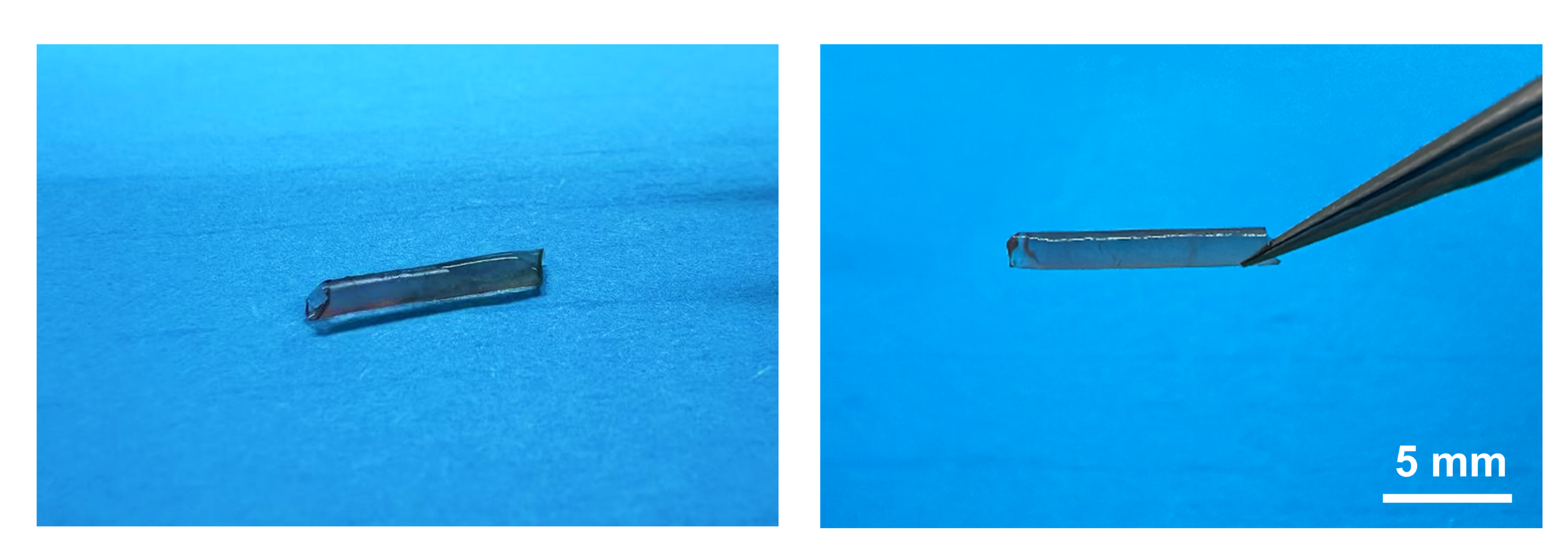


**Figure S11.** The self-supporting property of KLA coatings in a wet condition (Scale bar: 5 mm).


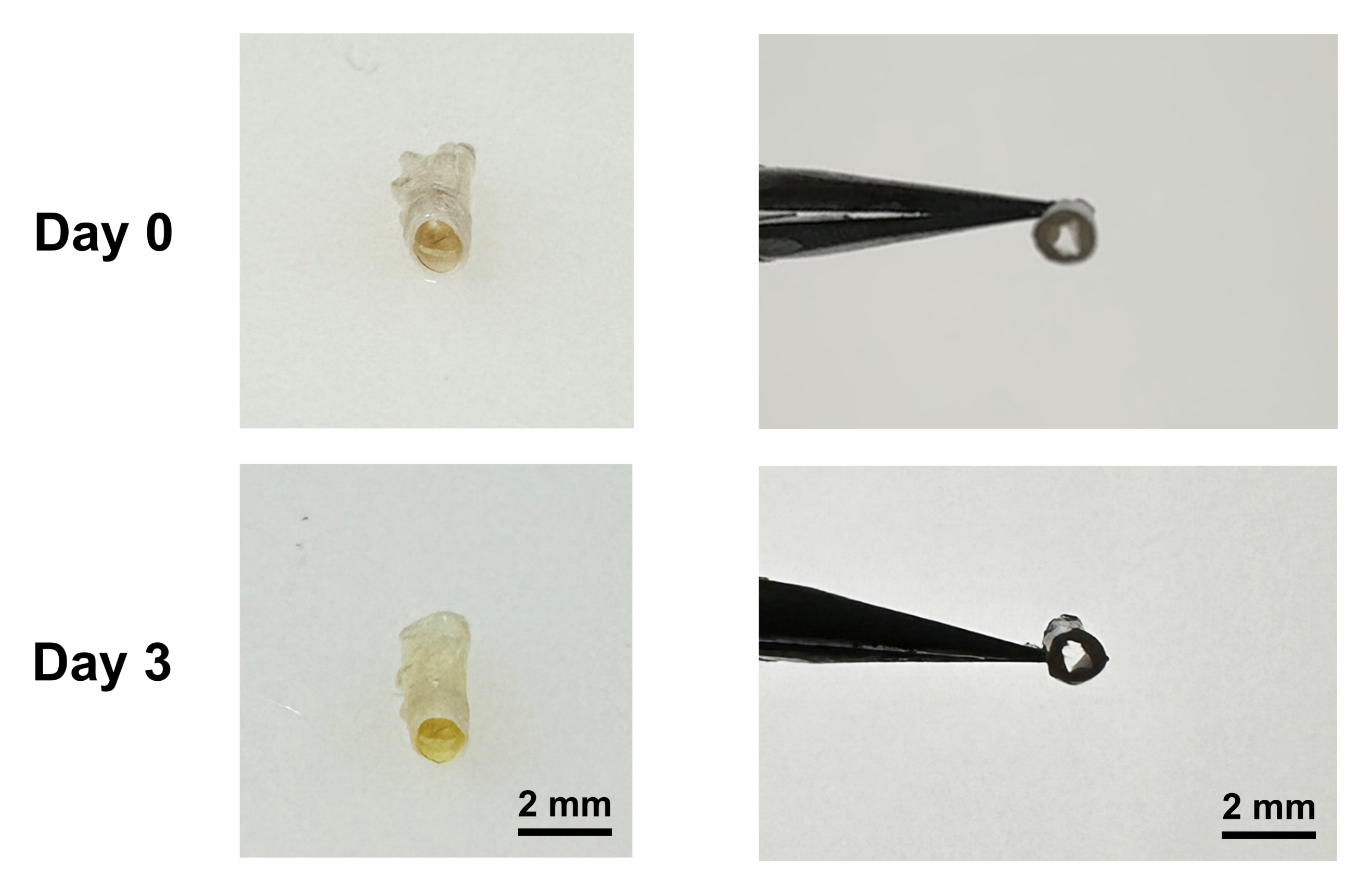


**Figure S12.** Digital photos of the blood vessel with PFD@BEAT coating before and after ex vitro circulation for 3 days (Scale bar: 2 mm).


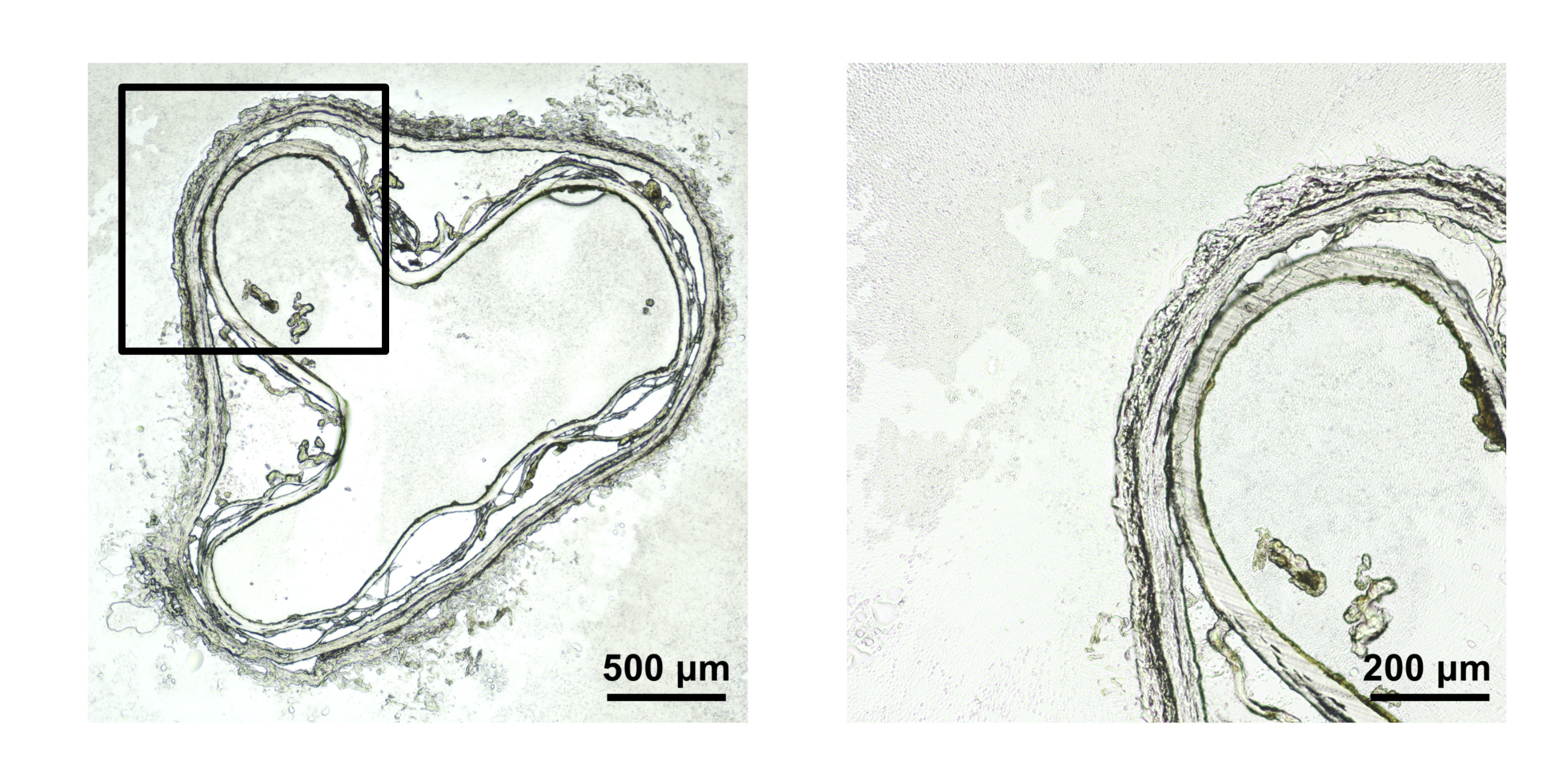


**Figure S13.** Sectional imaging following PFD@BEAT coating implantation (Scale bars: 500 μm and 200 μm).


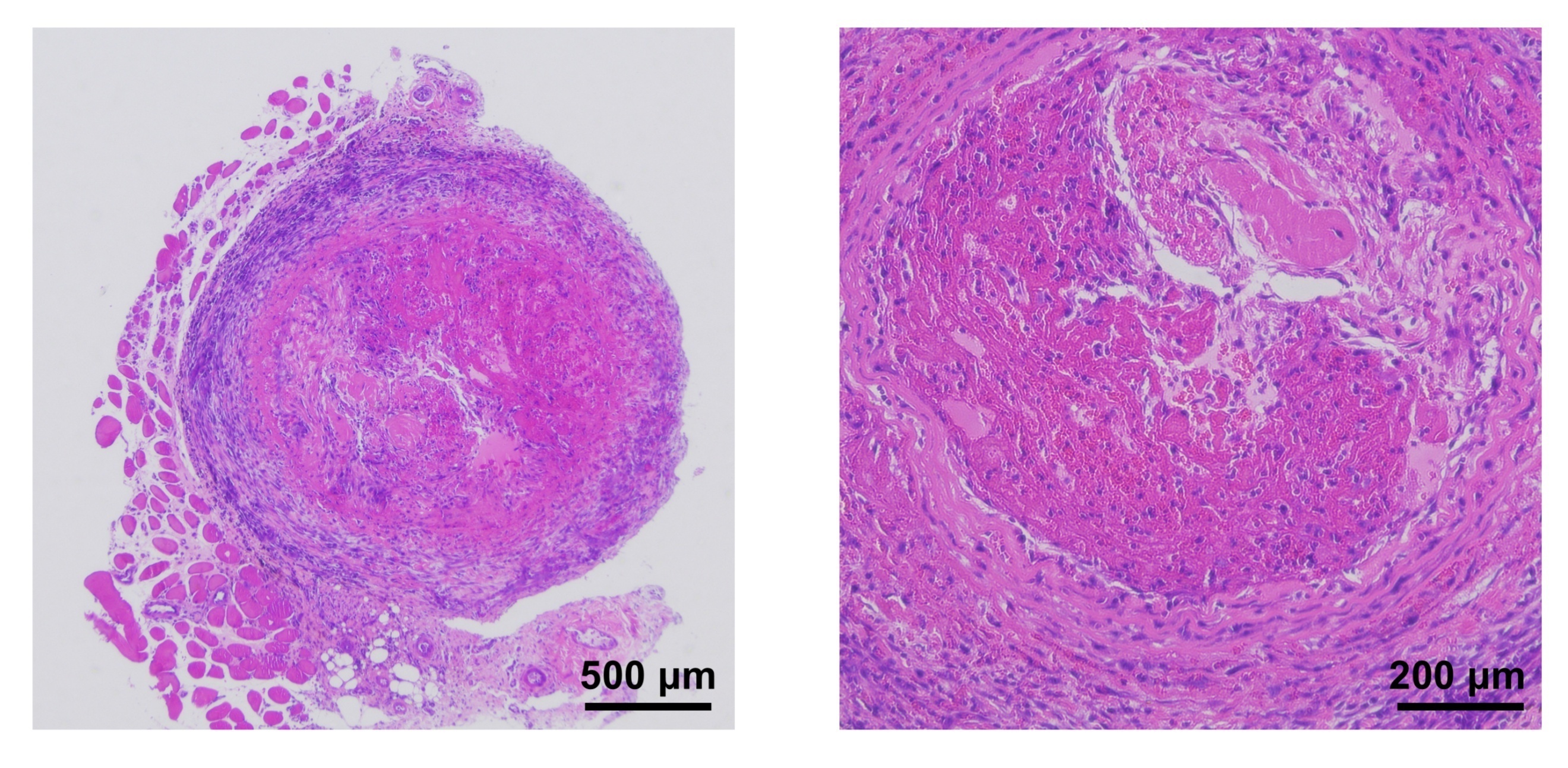


**Figure S14.** H&E staining images of vessels treated with PFD@HC-BEAT one day post-implantation (Scale bars: 500 μm and 200 μm).
